# An RXLR effector secreted by *Phytophthora parasitica* is a virulence factor and triggers cell death in various plants

**DOI:** 10.1111/mpp.12760

**Published:** 2018-11-22

**Authors:** Guiyan Huang, Zhirou Liu, Biao Gu, Hong Zhao, Jinbu Jia, Guangjin Fan, Yuling Meng, Yu Du, Weixing Shan

**Affiliations:** ^1^ State Key Laboratory of Crop Stress Biology for Arid Areas Northwest A&F University Yangling Shaanxi 712100 China; ^2^ College of Life Sciences Northwest A&F University Yangling Shaanxi 712100 China; ^3^ College of Plant Protection Northwest A&F University Yangling Shaanxi 712100 China; ^4^ Institute of Plant and Food Science, Department of Biology Southern University of Science and Technology Shenzhen 518055 China; ^5^ College of Agronomy Northwest A&F University Yangling Shaanxi 712100 China; ^6^ College of Horticulture Northwest A&F University Yangling Shaanxi 712100 China

**Keywords:** cell death, haustoria, *Phytophthora parasitica*, RXLR effector, virulence

## Abstract

RXLR effectors encoded by *Phytophthora* species play a central role in pathogen–plant interactions. An understanding of the biological functions of RXLR effectors is conducive to the illumination of the pathogenic mechanisms and the development of disease control strategies. However, the virulence function of *Phytophthora parasitica* RXLR effectors is poorly understood. Here, we describe the identification of a *P. parasitica* RXLR effector gene, *PPTG00121* (*PpE4*), which is highly transcribed during the early stages of infection. Live cell imaging of *P. parasitica* transformants expressing a full‐length PpE4 (E4FL)‐mCherry protein indicated that PpE4 is secreted and accumulates around haustoria during plant infection. Silencing of *PpE4* in *P. parasitica* resulted in significantly reduced virulence on *Nicotiana benthamiana*. Transient expression of *PpE4* in *N. benthamiana* in turn restored the pathogenicity of the *PpE4*‐silenced lines. Furthermore, the expression of *PpE4* in both *N. benthamiana* and *Arabidopsis thaliana* consistently enhanced plant susceptibility to *P. parasitica*. These results indicate that *PpE4* contributes to pathogen infection. Finally, heterologous expression experiments showed that *PpE4* triggers non‐specific cell death in a variety of plants, including tobacco, tomato, potato and *A. thaliana*. Virus‐induced gene silencing assays revealed that *PpE4*‐induced cell death is dependent on *HSP90*, *NPK* and *SGT1*, suggesting that PpE4 is recognized by the plant immune system. In conclusion, PpE4 is an important virulence RXLR effector of *P. parasitica* and recognized by a wide range of host plants.

## Introduction

*Phytophthora parasitica* shares the main features of most *Phytophthora* species; it is a soil‐borne pathogen with a wide host range (Meng *et al*., 2014). It causes tobacco black shank and is listed as one of the top 10 oomycete pathogens because of its scientific and economic importance (Kamoun *et al*., 2015). *P. parasitica* serves as a model oomycete pathogen, and its compatible interaction with the model plant *Arabidopsis thaliana* has been established (Attard *et al*., 2010; Wang Y *et al*., 2011). There have been fewer functional analyses of *P. parasitica* genes (Chang *et al*., 2015; Evangelisti *et al*., 2013; Gaulin *et al*., 2002; Khatib *et al*., 2004; Meng *et al*., 2015; Zhang *et al*., 2012), and these studies are far from sufficient to fully understand the biology, pathogenesis and plant interaction mechanisms of *P. parasitica*.

During the war between pathogens and hosts, plants have evolved two immune systems to defend against invaders: pathogen‐associated molecular pattern (PAMP)‐triggered immunity (PTI), mediated by pattern recognition receptors (PRRs), and effector‐triggered immunity (ETI), mediated by resistance (R) proteins that recognize avirulence (AVR) effectors (Dodds and Rathjen, 2010; Jones and Dangl, 2006). On perception of non‐self signals (PAMPs or effectors) from pathogens, plant cells activate a complicated signal transduction network. Although the signal transduction pathways implicated in PTI and ETI are different, the downstream cellular events are similar, including a series of cellular responses and also cell death (Dodds and Rathjen, 2010; Pedley and Martin, 2005; Peng *et al*., 2018). Although cell death induced by a number of *Phytophthora* RXLR effectors occurs independently of known R proteins, it is probably the result of plant recognition and related to components of the PTI or ETI pathway. Many genes involved in plant immune signalling are required for effector‐induced cell death. For example, *MEK2* is required for *Avh238*‐triggered cell death (Yang *et al*., 2017), *MEK2* and *WIPK* are involved in *Avh241*‐induced cell death (Yu *et al*., 2012), suppressor of G2 allele of *skp1* (*SGT1*) is required for the cell death activity of *PexRD2* (Oh *et al*., 2009) and *PITG_22798* (Wang H *et al*., 2017), a specific mitogen‐activated protein kinase (MAPK) cascade is responsible for *Pi_23226*‐induced cell death (Lee *et al*., 2018), and *SGT1*, *HSP90*, *RAR1* and MAPK cascades are required for *PvRXLR16*‐induced cell death (Xiang *et al*., 2017).

During the infection and colonization of plants, pathogens secrete numerous effectors to manipulate plant physiological processes and thereby suppress plant immunity and enhance plant susceptibility. Effectors usually possess dual activities, facilitating infection and triggering plant immunity during plant–microbial interactions (Kamoun, 2006; Kjemtrup *et al*., 2000; van’t Slot & Knogge, 2002). For example, the glycoside hydrolase 12 protein XEG1 is required for *Phytophthora sojae* virulence, but is also recognized as a PAMP and triggers cell death and plant immunity (Ma *et al*., 2015). Necrosis and ethylene‐inducing peptide 1 (Nep1)‐like proteins (NLPs), which are conserved virulence factors widespread in bacterial, oomycete and fungal pathogens, trigger host cell damage‐associated plant immunity and are also recognized as PAMPs (Bohm *et al*., 2014; Fellbrich *et al*., 2002; Ottmann *et al*., 2009; Qutob *et al*., 2006). Another classic example is the triggering of an *R* gene‐mediated hypersensitive response (HR) by AVR effectors that typically exert their virulence function on *R* gene‐absent plants (Kamoun, 2006). In addition to known AVR effectors, a few RXLR effectors, such as *PsAvh241* (Yu *et al*., 2012), *PsAvh238* (Wang Q *et al*., 2011; Yang *et al*., 2017) and *PITG_22798* (Wang H *et al*., 2017), possess virulence functions even though they induce immune response‐related cell death in plants.

RXLR effectors, which exist by the hundreds in each oomycete genome, are amongst the best‐characterized oomycete effectors (Baxter *et al*., 2010; Haas *et al*., 2009; Jiang *et al*., 2008; Tyler *et al*., 2006). In recent years, a large number of studies have been carried out to elucidate the biological functions of RXLR effectors from *Phytophthora infestans*, *P. sojae* and *Hyaloperonospora arabidopsidis* (Anderson *et al*., 2015; Sharpee and Dean, 2016; Wang Q *et al*., 2011; Whisson *et al*., 2016; Zheng *et al*., 2014). However, little is known about RXLR effectors from *P. parasitica*, except PSE1, which has been reported to alter the auxin content and to promote infection (Evangelisti *et al*., 2013). In addition, 172 candidate RXLR effectors have been identified recently in the *P. parasitica* genome, three of which suppress INF1‐induced cell death and enhance *P. parasitica* virulence (Dalio *et al*., 2017).

In this study, we investigated the virulence function of the *P. parasitica* RXLR effector gene *PpE4*. We found that *PpE4* is highly expressed during the early stages of infection and is secreted from haustoria. To evaluate the role of *PpE4* in *P. parasitica* pathogenicity, *PpE4*‐silenced transformants were created and analysed. These transformants showed a reduced ability to infect plants, and transient expression of *PpE4* in *Nicotiana benthamiana* restored pathogenicity. To further examine its contribution in the promotion of pathogen colonization, an inoculation assay was performed after transient or induced *in planta* expression of *PpE4*. Plants expressing *PpE4 *were more susceptible to *P. parasitica *infection. *PpE4* also triggered non‐specific cell death in a variety of plants in an *HSP90*‐, *NPK*‐ and *SGT1*‐dependent manner, which suggests that PpE4 is recognized by the plant immune system. Based on these results, we conclude that PpE4 is a virulence RXLR effector of *P. parasitica* and is recognized by a wide variety of host plants.

## Results

### *PpE4 *encodes a secreted RXLR effector and is highly expressed during the early phase of infection

**Figure 1:**
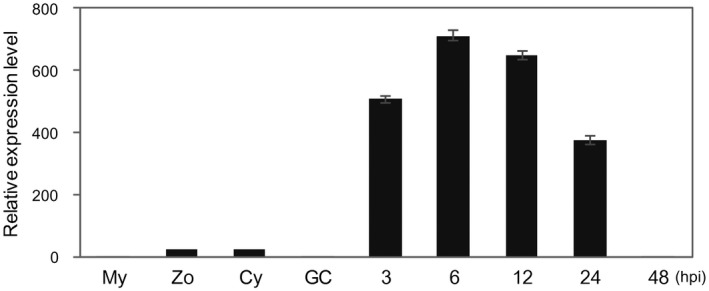
The *Phytophthora parasitica* RXLR effector gene *PpE4* is highly expressed during early plant infection. Reverse transcription‐quantitative polymerase chain reaction (RT‐qPCR) was used to quantify the relative *PpE4* transcript levels during different stages of *P. parasitica* development and infection. *Nicotiana benthamiana* leaves inoculated with *P. parasitica* zoospores were harvested at different hours post‐inoculation (hpi). Cy, cysts; GC, germinated cysts; My, *P. parasitica* mycelium grown in carrot broth; Zo, zoospores. The relative expression level of *PpE4* in mycelia was given a value of unity. Error bars represent the standard deviation (SD) of three biological replicates.

Using previous RNA‐sequencing (RNA‐seq) data (Jia *et al*., 2017), we identified a putative *P. parasitica* RXLR effector gene *PPTG_00121*, named *PpE4*, which was the most highly expressed RXLR effector gene during the infection of *Arabidopsis* roots (Fig. S1A, see Supporting Information). Over 70% of the total RXLR effector transcripts corresponded to *PpE4*, and the FPKM (fragments per kilobase million) value was over 8000 at 3–6 h post‐inoculation (hpi) of *P. parasitica* zoospores (Fig. S1A). To validate the expression profile of *PpE4* during development and plant infection, reverse transcription‐quantitative polymerase chain reaction (RT‐qPCR) (Bustin *et al*., 2009) was performed. The expression pattern of *P. parasitica PpE4* during *A. thaliana* root infection initiated with zoospores was consistent with RNA‐seq (Fig. S1B). During infection of *N. benthamiana* leaves, *PpE4 *transcripts were rapidly and strongly up‐regulated from 3 to 24 hpi, and then declined and became barely detectable at 48 hpi, which is similar to the observations in vegetative hyphae, zoospores, cysts and germinated cysts before infection (Fig. 1). Biotrophic growth of *P. parasitica* in *N. benthamiana* leaves was dominant before 24 hpi, followed by necrotrophic growth with significant cell death (Fig. S2, see Supporting Information). In conclusion, *PpE4* transcripts are strongly induced and predominantly accumulated during the biotrophic phase, at levels hundreds of times higher than those in the mycelium.

**Figure 2:**
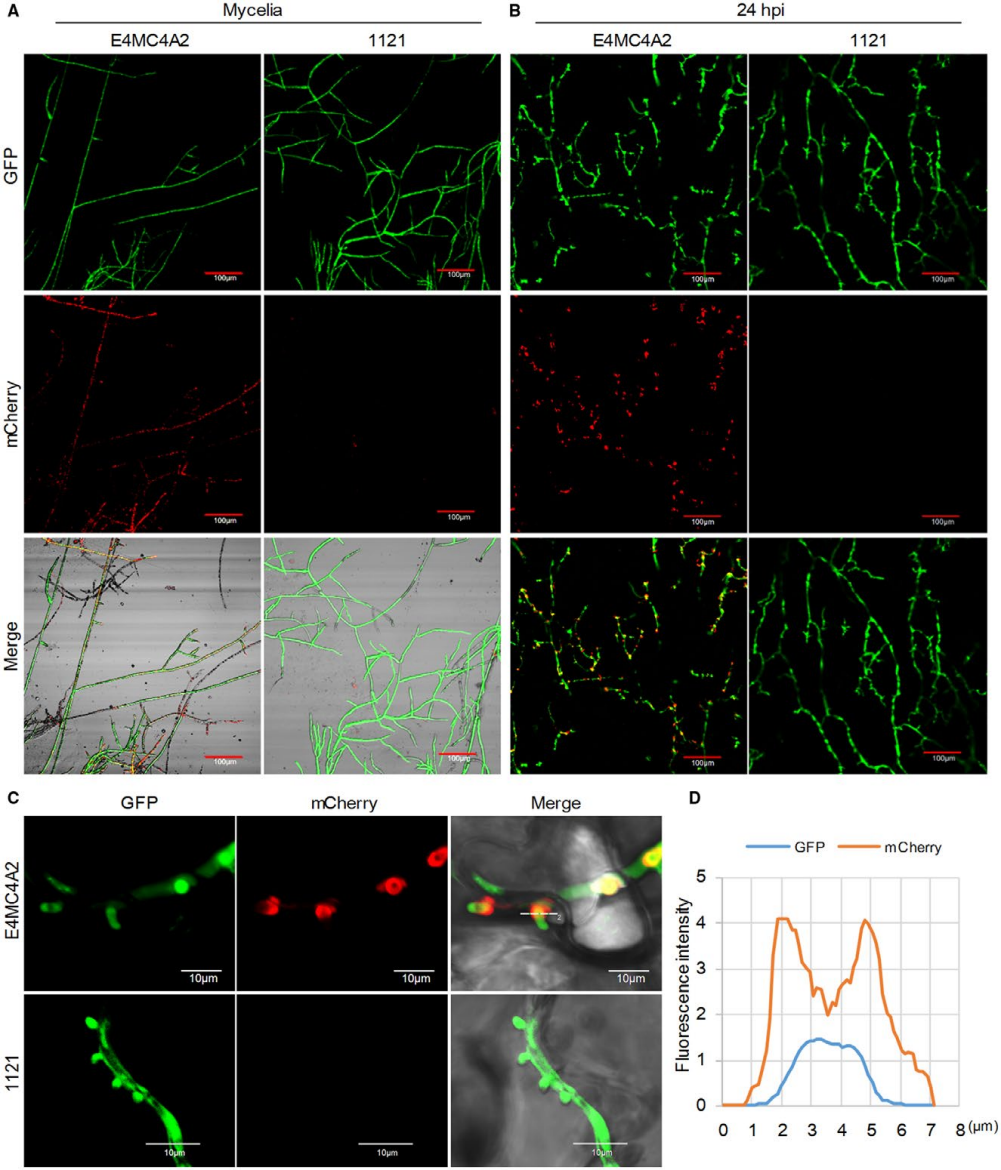
PpE4 accumulates around haustoria after secretion during *Phytophthora parasitica* infection. (A) Confocal images of mycelia cultured on 5% carrot juice agar medium. The red fluorescence was distributed throughout the mycelial cytoplasm of E4MC4A2 [a transformant expressing cytoplasmic green fluorescent protein (GFP) and full‐length PpE4 (E4FL)‐mCherry], but was not detected in strain 1121 (stably expressing cytoplasmic GFP). (B) *Nicotiana benthamiana* leaves infected with E4MC4A2 and 1121 were observed by confocal microscopy at 24 h post‐inoculation (hpi). A strong red fluorescence signal was highly accumulated in haustoria, but not in hyphae, during E4MC4A2 infection, whereas GFP fluorescence was evenly distributed in hyphae. No red fluorescence was observed in strain 1121. (C) A magnified lateral view of haustoria showing red fluorescence focused on the outside of the haustoria base and the GFP signal distributed throughout hyphae and haustoria. (D) The fluorescence intensities of GFP and mCherry across the haustorium indicated by the white line labelled ‘2’ in (C). Identical images were obtained from more than 10 haustoria in three independent biological replicates. [Colour figure can be viewed at wileyonlinelibrary.com]

To monitor the secretion of PpE4 during infection, a full‐length *PpE4* with its native signal peptide (*E4FL*)‐*mCherry* fusion construct was transformed into *P. parasitica* strain 1121, which stably expresses cytoplasmic green fluorescent protein (GFP), via polyethylene glycol (PEG)–CaCl_2_‐mediated transformation (Bottin *et al*., 1999). Six transformants showing a stable red fluorescence signal and one without were chosen for RT‐qPCR and western blot assays. High levels of *PpE4* transcripts and fusion proteins accumulated in vegetative mycelia of transformants, whereas no accumulation was observed in E4MC3N4 and strain 1121 (Fig. S3, see Supporting Information). The observation of transformant E4MC4A2 with a strong red fluorescence signal showed that the red fluorescence was evenly distributed in mycelia cultured *in vitro* (Fig. 2A), whereas it was highly enriched in haustoria during the infection of *N. benthamiana* leaves at 24 hpi (Fig. 2B). Further detailed observations and fluorescence intensity analyses of E4MC4A2 (Fig. 2C,D) and E4MC4A6 (Fig. S4, see Supporting Information) showed that the mCherry fluorescence signal accumulated outside the GFP fluorescent haustoria, mainly distributed around the haustorial neck, indicating that E4FL‐mCherry accumulates in the extrahaustorial matrix (EHMx) on secretion from haustoria. By contrast, there was no mCherry fluorescence in strain 1121, and GFP fluorescence was distributed evenly in vegetative and infection hyphae, without specific accumulation at haustoria (Fig. 2). This result is consistent with previous studies of *P. infestans* effectors AVR3a (Whisson *et al*., 2007), AVR2 (Gilroy *et al*., 2011), Pi04314 (Wang S *et al*., 2017), AVR4 and AVRblb1 (van Poppel, 2009), and *P. sojae* effector Avr1b (Liu *et al*., 2014).

### Silencing of *PpE4 *attenuates the pathogenicity of *P. parasitica*

**Figure 3:**
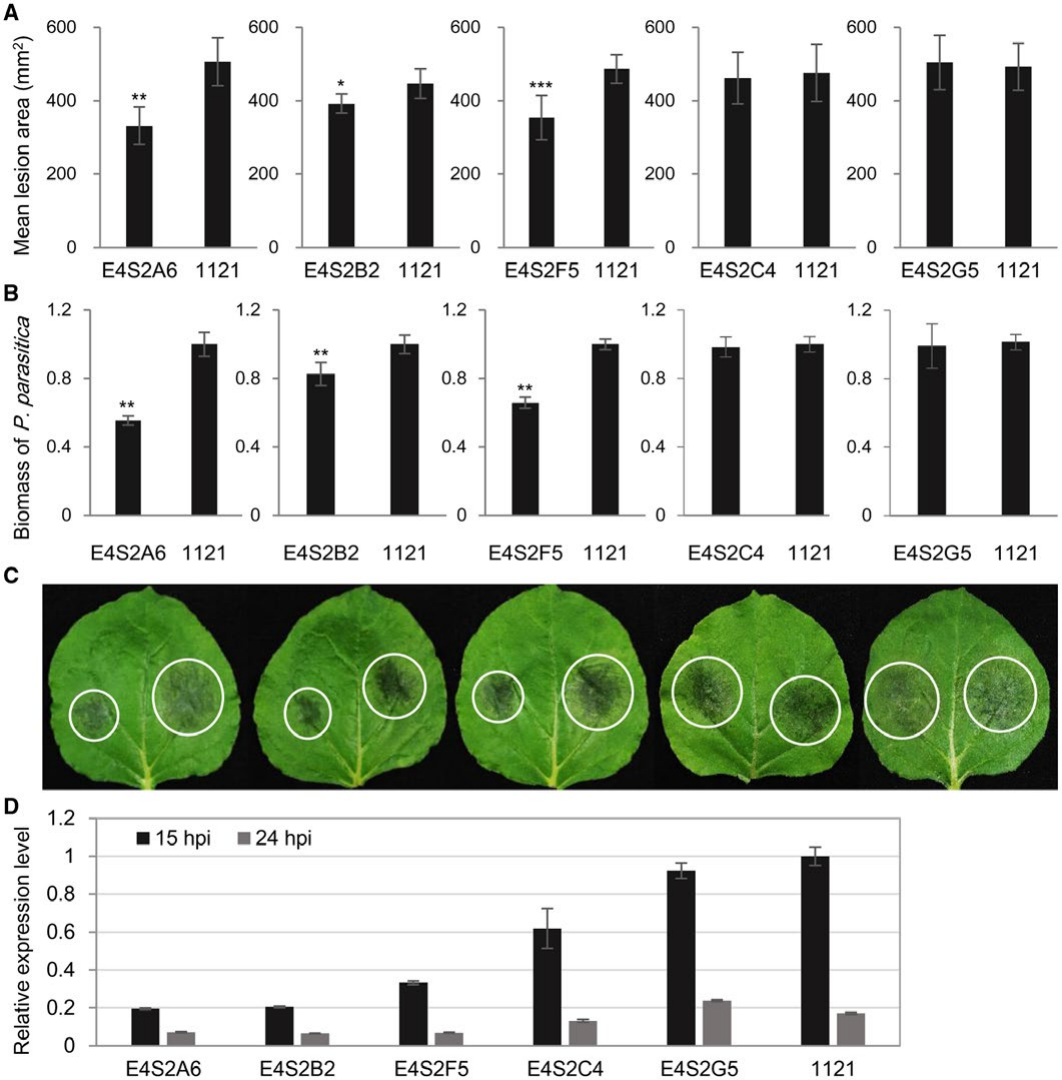
*PpE4*‐silenced *Phytophthora parasitica* transformants exhibit reduced pathogenicity. (A) Mean lesion areas of *Nicotiana benthamiana* leaves inoculated with *PpE4*‐silenced transformants and the control strain 1121 at 48 h post‐inoculation (hpi)*.* The transformant and the control strain were inoculated on opposite halves of an *N. benthamiana* leaf. Error bars represent the standard deviation (SD) of 15 leaves, and asterisks denote significant differences from control strain 1121 (two tailed *t*‐test: *P < 0.05; **P < 0.01; ***P < 0.001). (B) Biomass of *P. parasitica* on *N. benthamiana* leaves determined by quantitative polymerase chain reaction (qPCR). Bars represent *PpUBC* levels relative to *NbF*‐*box* levels with SD of three biological replicates. Asterisks denote significant differences from control strain 1121 (two tailed *t*‐test: **P < 0.01). (C) Representative inoculated leaves. White circles outline the water‐soaked lesions. Similar results were obtained from more than three independent experiments with about 15 leaves for each experiment. (D) *PpE4* expression was restored in two transformants E4S2C4 and E4S2G5, whose virulence was not reduced. The subcultured transformants were inoculated onto *N. benthamiana* leaves and sampled at 15 and 24 hpi. Reverse transcription (RT)‐qPCR was used to determine the *PpE4* silencing level. The expression level of *PpE4* in strain 1121 sampled at 15 hpi was given a value of unity. Error bars represent the SD of three biological replicates. Two independent experiments were performed with similar results. [Colour figure can be viewed at wileyonlinelibrary.com]

Inoculation analysis revealed that constitutive expression of *E4FL‐mCherry* reduces colonization by *P. parasitica* (Fig. S5, see Supporting Information). To investigate the potential virulence function of *PpE4* in *P. parasitica* pathogenesis, we generated co‐silencing transformants as described previously (Meng *et al*., 2015). A hairpin structure derived from a segment of *GFP* fused with a segment of *PpE4* was constructed and introduced into *P. parasitica* strain 1121 (Fig. S6A, see Supporting Information). Because both *GFP *and *PpE4 *were targeted, *PpE4* expression was more likely to be decreased in transformants with a significantly reduced GFP signal. A total of 173 independent transformants were generated, and 19 with normal colony morphology showed decreased GFP fluorescence, a frequency consistent with previous reports (Meng *et al*., 2015; Zhang *et al*., 2012). RT‐qPCR experiments revealed that five of the 19 candidate transformants had obviously reduced *PpE4* expression at 24 hpi compared with strain 1121 (Fig. S6). Further pathogenicity analysis showed that three silenced lines (E4S2A6, E4S2B2 and E4S2F5) produced significantly smaller lesions and less hyphal biomass compared with strain 1121, whereas the virulence of the other two lines (E4S2C4 and E4S2G5) was almost unaffected (Fig. 3A–C). We further confirmed the expression of *PpE4* in these transformants after a series of subcultures. The results showed that the expression level of *PpE4* in infected *N. benthamiana* leaves at 15 hpi and 24 hpi remained silenced in transformants E4S2A6, E4S2B2 and E4S2F5, but partially recovered in E4S2C4 and totally recovered in E4S2G5 (Fig. 3D), which was consistent with the results of the pathogenicity assay. Therefore, stable silencing of *PpE4* led to the attenuated pathogenicity of *P. parasitica* and restored target gene expression, suggesting that *PpE4* is important to *P. parasitica*.

### Transient expression of *PpE4 in planta *restores the pathogenicity of *PpE4*‐silenced transformants

**Figure 4:**
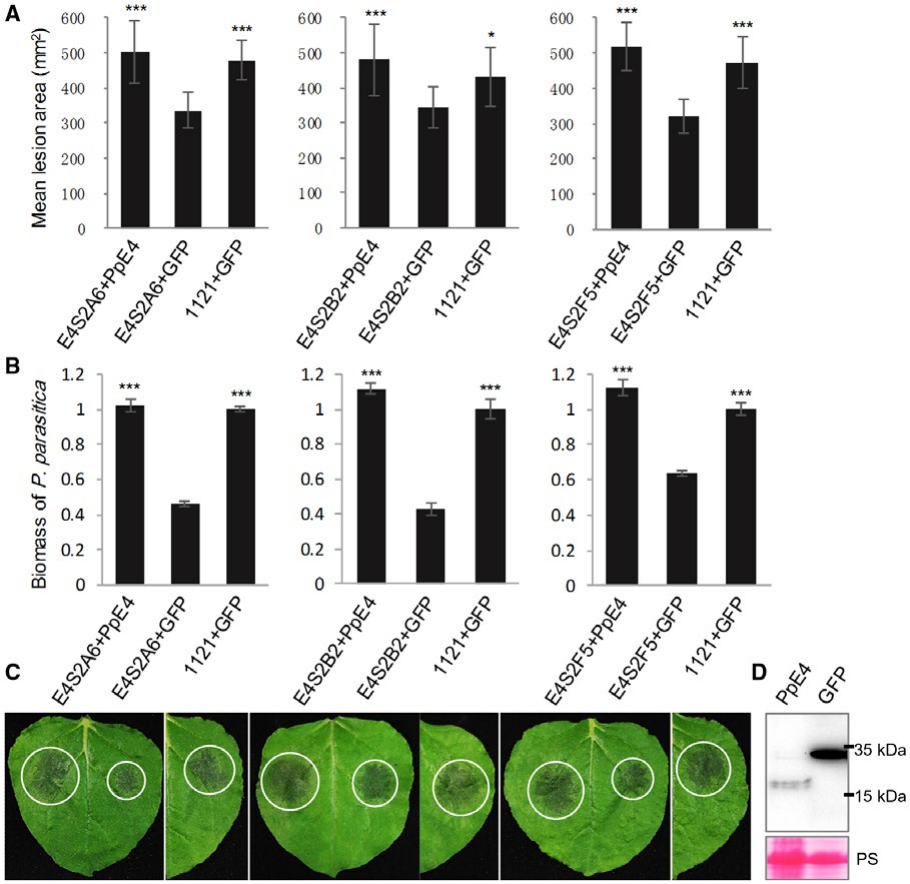
The pathogenicity of *PpE4*‐silenced *Phytophthora parasitica* lines is restored by the transient expression of *PpE4 in planta*. *PpE4* and *GFP* were transiently expressed by agroinfiltration in *Nicotiana benthamiana* leaves 1 day before inoculation [optical density at 600 nm (OD_600_) = 0.01]. (A) The lesions formed after the inoculation of *PpE4*‐silenced lines onto *PpE4*‐expressing leaves were almost the same size as those formed after the inoculation of 1121 onto *GFP*‐expressing leaves, whereas the lesions formed by silenced lines inoculated onto *GFP*‐expressing leaves were significantly smaller. Error bars represent the standard deviation (SD) of 15 leaves, and asterisks denote significant differences from the control group (two‐tailed *t*‐test: *P < 0.05; ***P < 0.001). (B) Biomass of *P. parasitica* on *N. benthamiana* leaves was determined by quantitative polymerase chain reaction (qPCR). Bars represent *PpUBC* levels relative to *NbF*‐*box* levels with SD of three biological replicates. Asterisks denote significant differences from silenced lines inoculated onto *GFP*‐expressing leaves (two‐tailed *t*‐test: ***P < 0.001). (C) Representative inoculated leaves. White circles outline the water‐soaked lesions. (D) Protein accumulation detected by western blot using anti‐Flag antibody. Protein loading is indicated by Ponceau stain (PS). Similar results were obtained from three independent experiments with more than 15 leaves inoculated for each group in each experiment. [Colour figure can be viewed at wileyonlinelibrary.com]

To verify that the virulence attenuation of the *PpE4*‐silenced lines is caused by *PpE4* silencing, three silenced lines were inoculated onto mature *PpE4*‐expressing (intracellular expression without signal peptide) or *GFP*‐expressing leaves, and strain 1121 was inoculated onto *GFP*‐expressing leaves. The *PpE4*‐silenced lines inoculated onto *PpE4*‐expressing leaves formed significantly larger lesions than those inoculated onto *GFP*‐expressing leaves, whereas there was no difference between the size of the lesions on *PpE4*‐expressing leaves and those of the control group (1121 inoculated on *GFP*‐expressing leaves) (Fig. 4A–C). Western blot showed that PpE4 and GFP proteins were stably accumulated under low agroinfiltration concentration [optical density at 600 nm (OD_600_) = 0.01] (Fig. 4D). This indicates that *in planta* expression of *PpE4* is able to restore the virulence of *PpE4*‐silenced lines to wild‐type levels. In conclusion, *PpE4* positively contributes to the pathogenicity of *P. parasitica*.

### *PpE4 *enhances plant susceptibility to *P. parasitica*

**Figure 5:**
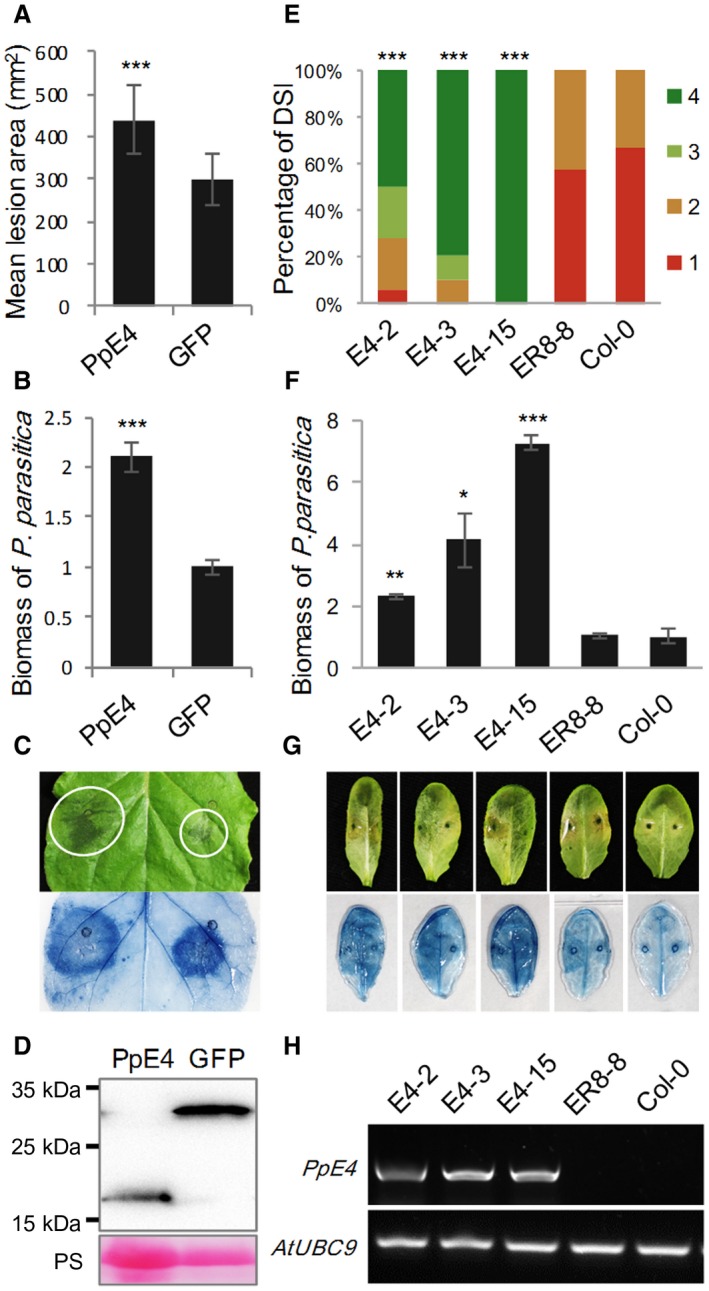
Heterologous expression of *PpE4* renders *Nicotiana benthamiana* and *Arabidopsis* more susceptible to *Phytophthora parasitica* infection. (A) Mean lesion areas were measured at 48 h post‐inoculation (hpi). *Agrobacterium tumefaciens* strains carrying *PpE4* or *GFP* [optical density at 600 nm (OD_600_) = 0.01] were infiltrated into different sides of the same leaf, 1 day before inoculation of strain 1121. Error bars represent the standard deviation (SD) of 15 leaves, and asterisks denote significant differences from the green fluorescent protein (GFP) control (two‐tailed *t*‐test: ***P < 0.001). (B) Quantification of *P. parasitica* biomass in infected *N. benthamiana* leaves. Bars represent *PpUBC* levels relative to *NbF*‐*box* levels with SD of three biological replicates. Asterisks denote significant differences from the GFP control (two‐tailed *t*‐test: ***P < 0.001). (C) A typical leaf photographed and stained by trypan blue. White circles outline the water‐soaked lesions. (D) Protein accumulation was determined at 3 days post‐infiltration (dpi) by western blot using anti‐Flag antibody. Protein loading is indicated by Ponceau stain (PS). Similar results were obtained from three independent experiments with about 15 leaves for each experiment. (E) Disease severity index (DSI) from grade 1 to grade 4 was recorded at 48 hpi. Homozygous transgenic plants expressing β‐estradiol‐inducible 3×*Flag‐PpE4 *(E4‐2, E4‐3 and E4‐15), an empty vector pER8 transgenic plant (ER8‐8) and wild‐type Col‐0 were injected with 10 μM 17‐β‐estradiol, 12 h before inoculation of strain 1121. Asterisks represent significant differences from Col‐0 (Wilcoxon rank‐sum test: ***P < 0.001). (F) Biomass of *P. parasitica* on *Arabidopsis* leaves. Bars represent *PpUBC* levels relative to *AtUBC* levels with SD of five biological replicates. Asterisks denote significant differences from Col‐0 (two‐tailed *t*‐test: *P < 0.05; **P < 0.01; ***P < 0.001). (G) Disease symptoms of representative leaves. Trypan blue stain was used to highlight the infection hyphae in colonized leaves. (H) Verification of *PpE4* expression 12 h after injection of 10 μM 17‐β‐estradiol using semi‐quantitative polymerase chain reaction (PCR). Similar results were obtained from three independent experiments with about 25 leaves for each experiment. [Colour figure can be viewed at wileyonlinelibrary.com]

To further determine whether *PpE4* contributes to *P. parasitica* colonization *in planta*, inoculation was performed onto *N. benthamiana* leaves expressing mature PpE4 on one half and GFP on the other. The lesions and *P. parasitica* biomass on the *PpE4*‐expressing halves were significantly larger than those on the control (Fig. 5A–C). Stable accumulation of PpE4 and GFP proteins *in planta* was detected by western blot (Fig. 5D). These results suggest that the transient expression of *PpE4* renders *N. benthamiana* more susceptible to *P. parasitica*.

We also examined the contribution of *PpE4* in the *Arabidopsis*–*P. parasitica* pathosystem. Chemically inducible transgenic *Arabidopsis* lines in which the expression of mature *PpE4* is strictly regulated by estradiol were constructed (Zuo *et al*., 2000). Wild‐type Col‐0 and empty vector pER8 transgenic plants were used as controls. The rosette leaves from three homozygous transgenic lines expressing *PpE4* and control plants were infiltrated with 10 μM 17‐β‐estradiol to induce *PpE4* expression, 12 h before inoculation of *P. parasitica* zoospores. The disease index statistic indicated that the transgenic plants expressing *PpE4* were more susceptible than the controls to *P. parasitica* infection, and the pathogen biomass in these plants was significantly higher than that in the control plants (Fig. 5E–G). Semi‐quantitative PCR showed that *PpE4* was expressed on injection of 17‐β‐estradiol, whereas there was no *PpE4* expression in control plants receiving the same treatment (Fig. 5H). These results demonstrate that *PpE4* facilitates *P. parasitica* infection.

### *PpE4* triggers cell death in various plants

**Figure 6:**
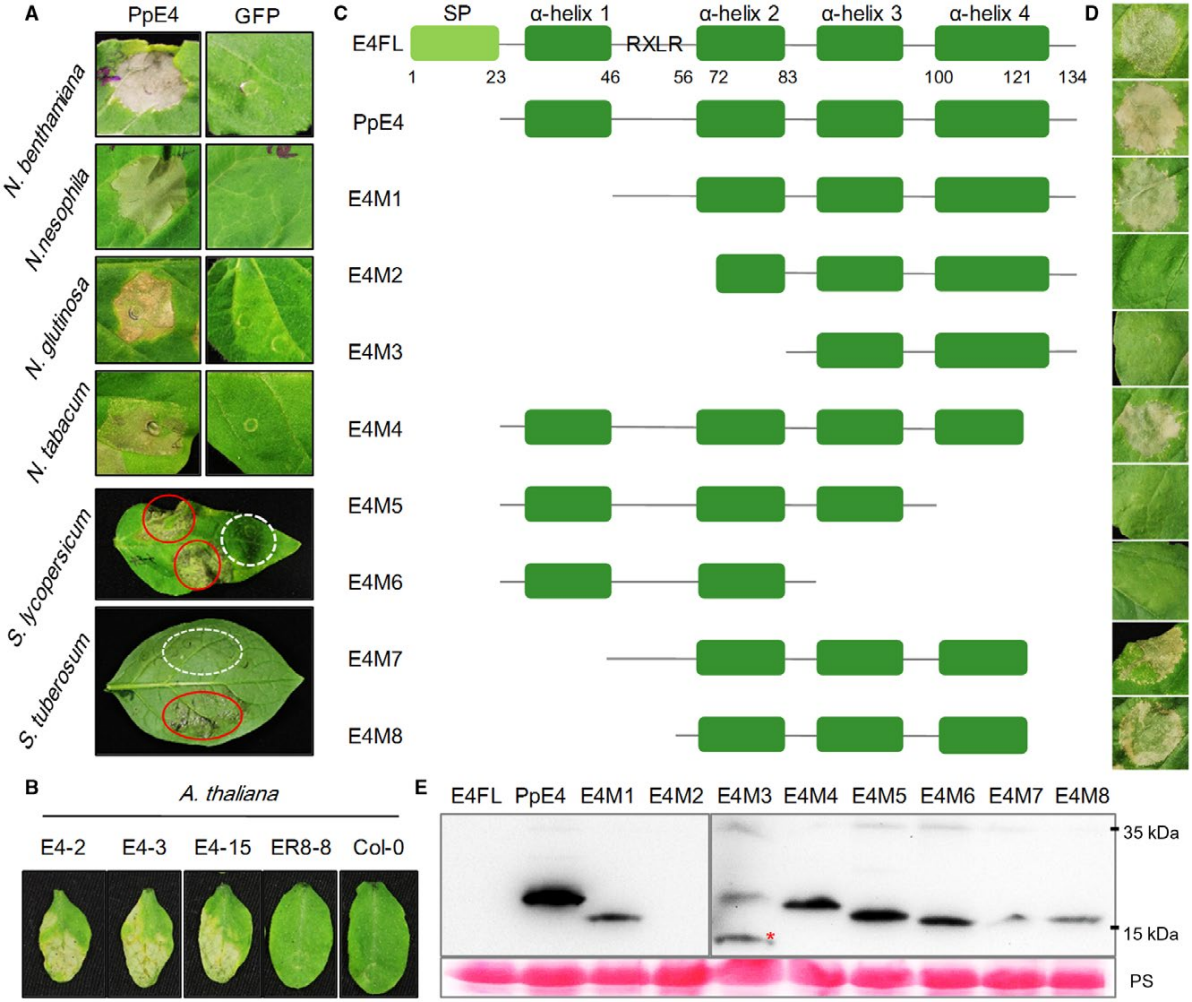
Analysis of cell death triggered by *PpE4*. (A) Cell death phenotype induced by *PpE4* in Solanaceae plants. *Agrobacterium tumefaciens* carrying *PpE4* [optical density at 600 nm (OD_600_) = 0.4] was infiltrated into the leaves of *Nicotiana benthamiana*, *N. nesophila*, *N. glutinosa*, *N. tabacum* cv. Florida 301, *Solanum lycopersicum* and *S. tuberosum*. Photographs were taken at 5 days post‐infiltration (dpi) for *Nicotiana* species and 8 dpi for *Solanum* species. Red circles represent the *PpE4*‐expressing areas, and white broken circles represent the *GFP*‐expressing areas. (B) Cell death symptoms triggered by *PpE4* in *Arabidopsis*. Leaves of transgenic *Arabidopsis *plants harbouring pER8::3×*Flag*‐*PpE4* or the empty vector and Col‐0 were injected with 10 μM 17‐β‐estradiol. Photographs were taken after 5 days. (C) Schematic diagrams of the protein secondary structures of the PpE4 deletion mutants. (D) Cell death symptoms in *N. benthamiana* leaves expressing PpE4 deletion mutants. Photographs were taken at 5 dpi. (E) Western blot detection of PpE4 deletion proteins using anti‐Flag antibody. The red asterisk indicates a protein band of the correct size. Protein loading is indicated by Ponceau stain (PS). Similar results were obtained from three independent experiments. [Colour figure can be viewed at wileyonlinelibrary.com]

When *PpE4* was intracellularly expressed in *N. benthamiana* leaves by agroinfiltration, it triggered cell death at 3 days post‐infiltration (dpi) (Fig. 6A). To investigate whether this cell death is species specific, *PpE4* was transiently expressed in several Solanaceae plants, including three tobacco species, tomato and potato. We found that *PpE4* triggered cell death in all tested Solanaceae plants (Fig. 6A). No *Arabidopsis *transgenic plants were recovered when the 35S promoter was used to drive *PpE4* expression, implying that *PpE4* is lethal to *Arabidopsis* cells. Using estradiol‐inducible transgenic plants, we found that *PpE4* triggered cell death in *Arabidopsis* leaves 4 days after induction by 17‐β‐estradiol, whereas no cell death occurred in the control plants (empty vector pER8 transgenic plants and Col‐0) (Fig. 6B). These results indicate that *PpE4* triggers non‐specific cell death in a variety of plants.

According to the protein secondary structure predicted by Phyre2 (Kelley *et al*., 2015), PpE4 contains four α‐helices downstream of the signal peptide (Fig. S7, see Supporting Information). To identify which domains are crucial for cell death‐inducing activity, we successively deleted the α‐helices to construct a series of deletion mutants and transiently expressed them in *N. benthamiana* (Fig. 6C–E). E4FL with its native signal peptide could also induce cell death. However, cell death occurred more slowly and more weakly in comparison with that of the mature protein. As shown in Fig. 6, E4M1, E4M4, E4M7 and E4M8 still maintained cell death‐inducing activity, which indicates that the first α‐helix, the RXLR motif and the last 13 amino acids are not required for cell death induction. Deletion of the RXLR‐DEER domain as well as the second α‐helix abolished cell death‐inducing ability. As the DEER motif is in α‐helix 2, its deletion may destroy the structure of α‐helix 2. Moreover, E4M5 and E4M6 were unable to induce cell death, which indicates that α‐helix 3 and part of α‐helix 4 are necessary for cell death induction (Fig. 6C,D). In short, E4M8, with residues 56–121, is sufficient to maintain the integrity of the protein tertiary structure and to trigger cell death.

### *PpE4*‐induced cell death requires *HSP90*, *NPK *and *SGT1*

**Figure 7:**
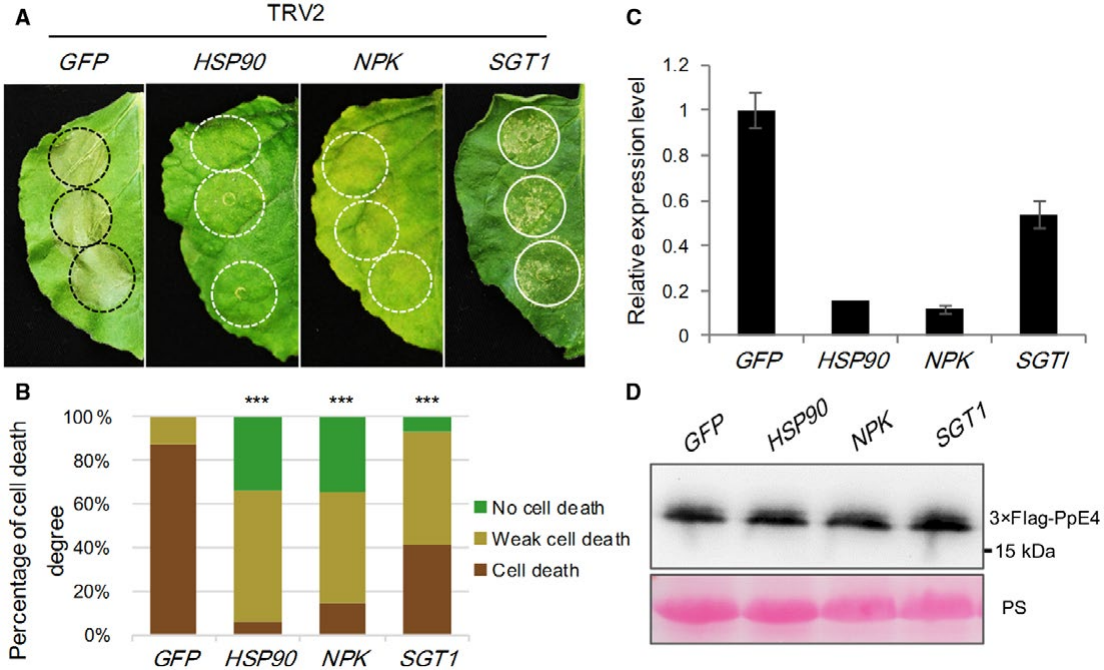
*HSP90*, *NPK* and *SGT1* are involved in *PpE4*‐induced cell death. (A) Representative images of *PpE4*‐induced cell death in silenced *Nicotiana benthamiana *leaves at 5 days post‐infiltration (dpi). *PpE4* was transiently expressed in the upper leaves of silenced plants at 16–20 dpi of TRV constructs. (B) Quantification of cell death in *N. benthamiana *leaves scored at 5 dpi. The degree of cell death was divided into three levels: no cell death, weak cell death and complete cell death. Asterisks indicate significant differences from *GFP*‐silenced plants (Wilcoxon rank‐sum test: ***P < 0.001). (C) Relative expression levels of *HSP90*, *NPK* and *SGT1* transcripts in corresponding virus‐induced gene silencing (VIGS)‐treated plants determined by reverse transcription‐quantitative polymerase chain reaction (RT‐qPCR). Error bars represent the standard deviation (SD) of three biological replicates. (D) Detection of PpE4 protein accumulation in silenced leaves using the anti‐Flag antibody. Protein loading is indicated by Ponceau stain (PS). Similar results were obtained from more than three independent experiments with 10 plants for each TRV construct. [Colour figure can be viewed at wileyonlinelibrary.com]

Cell death induced by a number of pathogen effectors is considered to be the outcome of recognition by the plant immune system, either PTI or ETI, involving a variety of receptors and signal transduction pathways (Lee *et al*., 2018; Wang H et al., 2017; Wang Q *et al*., 2011; Xiang *et al*., 2017; Yang *et al*., 2017; Yu *et al*., 2012). To determine which signalling pathway is involved in *PpE4*‐induced cell death, virus‐induced gene silencing (VIGS) was used to silence a series of genes in *N. benthamiana*, including genes responsible for R protein function, such as *HSP90*, *SGT1* and *RAR1* (Kanzaki *et al*., 2003; Takahashi *et al*., 2003; Zhang *et al*., 2004), genes associated with the activation of the TIR‐NB‐LRR (Toll/interleukin‐1 receptor, nucleotide binding and leucine‐rich repeat) and CC‐NB‐LRR (coiled coil, nucleotide binding and leucine‐rich repeat) R proteins, *EDS1* and *NDR1* (Knepper *et al*., 2011; Oh *et al*., 2014), respectively, the receptor‐like kinases *BAK1* and *SOBIR1* (Chaparro‐Garcia *et al*., 2011; Liebrand *et al*., 2013, 2014), the transcription factors *MYB1* and *WRKY3*, and the MAPK cascade genes *NPK*, *MEK1*, *MEK2* and *SIPK* (Jin *et al*., 2002; Liu *et al*., 2004b). Cell death was scored after transient expression of *PpE4* in these silenced plants. *PpE4*‐induced cell death was almost abolished in *HSP90*‐ and *NPK*‐silenced plants, and significantly attenuated in *SGT1*‐silenced plants, compared with *GFP*‐silenced plants (Fig. 7A,B). Western blot assay showed that the PpE4 protein was stably accumulated in the silenced plants (Fig. 7D). In addition, cell death was slightly, but significantly, compromised in *BAK1*‐silenced plants, whereas cell death was not affected in plants with silenced expression of the other genes (Fig. S8A,B, see Supporting Information). RT‐qPCR assays confirmed that there was a significant reduction in the transcript levels of the targeted genes in silenced plants compared with the levels in *GFP*‐silenced plants (Figs 7C and S8C). In summary, *HSP90*, *NPK* and *SGT1* are required for *PpE4*‐induced cell death.

## Discussion

### PpE4 is highly induced during infection and secreted from haustoria

As reported previously, functionally important RXLR effectors are usually induced during infection stages, but only a few RXLR effectors, which contribute the vast majority of RXLR effector transcripts, are considered to be crucial for pathogen pathogenicity (Wang Q *et al*., 2011). Here, we found that the effector gene *PpE4* is highly up‐regulated, accounting for more than 70% of the total RXLR effector transcripts during the early stages of infection (Figs 1 and S1). The relative expression of *PpE4* in the transformants was hundreds of times higher than that in strain 1121 in vegetative mycelia. However, only three transformants exhibited a slightly higher expression level than strain 1121 at 36 hpi (Fig. S3B,C), indicating that *PpE4* is extremely highly transcribed during infection and is difficult to be over‐expressed artificially by *Hsp70* or *Ham34* promoter during infection. This implies that *PpE4* plays a critical role during *P. parasitica* infection. RXLR effectors usually accumulate in the EHMx and are especially concentrated at the haustorial neck after secretion (Gilroy *et al*., 2011; Liu *et al*., 2014; van Poppel, 2009; Wang S *et al*., 2017; Whisson *et al*., 2007). In this study, we demonstrated that E4FL‐mCherry fusion protein accumulates substantially in the EHMx after secretion from haustoria during infection, even though its expression is driven by constitutive promoters (Figs 2, S3 and S4).

### *PpE4* contributes to infection even though it triggers cell death in plants

In virulence assays of *PpE4*‐silenced transformants, three stable silenced lines showed attenuated pathogenicity that could be restored by the transient expression of *PpE4 in planta*. However, the virulence of two other unstable transformants, E4S2C4 and E4S2G5, was not attenuated, with *PpE4* expression being initially silenced, but restored after a series of subcultures (Figs 3 and 4). These results indicate that stable silencing of *PpE4* affects the pathogenicity of *P. parasitica*. The restored expression of *PpE4* in the silenced transformants and its low frequency of co‐silencing with GFP suggest its importance and tightly regulated expression in *P. parasitica*, similar to a previous report (Meng *et al*., 2015). We also found that *E4FL*‐*mCherry*‐expressing transformants displayed decreased virulence (Fig. S5). The virulence reduction may be attributed to PpE4 recognition by the plant immune system. Considering that *PpE4* is transcribed in small amounts in mycelia and zoospores, it is likely that the constitutive overexpression of *PpE4* during the pre‐infection stage disrupts its original expression pattern and affects its pathogenicity. Similarly, the premature expression of *Avh238* also affects the ability of *P. sojae* to infect plants; thus, the timing of effector expression is crucial for pathogenicity (Wang Q *et al*., 2011).

In addition to its contribution to infection, we also found that *PpE4* triggers non‐specific cell death in two major eudicots (Fig. 6). Cell death plays a vital, but ambiguous, role in plant–pathogen interactions, especially those involving biotrophic and hemibiotrophic pathogens. Hemibiotrophs require living cells to establish colonization, and cell death is not preferred at the early stages of infection. During rapid expansion in plants, the invaders induce host cell death to facilitate the transition from biotrophy to necrotrophy (Qutob *et al*., 2002). However, cell death induced by the recognition of PAMPs or AVR effectors emerging at the very beginning of infection usually abolishes pathogen invasion. Therefore, the timing and intensity of cell death are under sophisticated regulation during plant–pathogen interactions: that which controls cell death wins (Coll *et al*., 2011; Kabbage *et al*., 2013).

Many RXLR effectors have been demonstrated to promote pathogen colonization when transiently expressed in plants, such as AVR1 (Du *et al*., a,b), PexRD2 (King *et al*., 2014), PITG_22798 (Wang H *et al*., 2017), Avh241 (Yu *et al*., 2012) and Avh238 (Yang *et al*., 2017). However, these effectors could also induce HR when detected by corresponding R proteins or recognized by unknown mechanisms in plants. To eliminate the influence of cell death during the inoculation process, we reduced the concentration of the *Agrobacterium tumefaciens* suspension to an OD_600_ value of 0.01 to delay and weaken cell death (Wang H *et al*., 2017). As a result, *PpE4* enhanced infection when transiently or stably expressed in plants, in spite of its cell death‐inducing activity (Figs 5 and 6). Three hypotheses may explain this result. First, although *PpE4* is highly transcribed at the early stages of infection, the accumulation of PpE4 protein in plant cells via translocation from the pathogen may be insufficient to induce cell death under natural conditions. Second, considering the cases of Avh241 and Avh238, where cell death could be suppressed by other immediate‐early expressed effectors (Wang Q *et al*., 2011), we suspect that *PpE4*‐induced cell death may be suppressed by other cooperative effectors. Thus, *PpE4* possibly manifests its virulence function and enhances colonization when its cell death activity is blocked. Finally, it is possible that cell death occurs just in time to promote the transition into necrotrophy, enabling an earlier occurrence of the necrotrophic phase. In this situation, cell death triggered by its intracellular expression is beneficial to pathogen infection, making it a virulence factor. In any case, *PpE4* exhibits dual functions: it contributes to *P. parasitica* virulence, whilst triggering recognition‐related cell death in the host plant.

### *PpE4*‐triggered cell death may be related to plant recognition

In this study, we applied VIGS technology to demonstrate that *PpE4*‐induced cell death requires *HSP90*, *NPK* and *SGT1 *(Fig. 7). As reported previously, HSP90 often forms a complex with its co‐chaperones RAR1 and SGT1 to maintain the function of NB‐LRR proteins (Kadota *et al*., 2010; Shirasu, 2009). In addition to being involved in R3a‐AVR3a‐mediated HR and INF1‐triggered cell death (Bos *et al*., 2006; Chapman *et al*., 2014; Kanzaki *et al*., 2003), HSP90 and SGT1 are required in both *N‐* and *Rx*‐mediated defence responses against viruses (Boter *et al*., 2007; Liu *et al*., 2004a; Lu *et al*., 2003). However, only SGT1 is required for PITG_22798‐ and Rpiblb2‐AVRblb2‐triggered HR (Oh *et al*., 2014; Wang H *et al*., 2017). *NPK1* is the *Nicotiana* homologue of human *MEKK1* and encodes a MAP kinase kinase kinase that is involved in responses mediated by the resistance genes *N*, *Bs2* and *Rx* (Jin *et al*., 2002; Liu *et al*., 2004b; Soyano *et al*., 2003). The fact that *HSP90*, *NPK* and *SGT1 *are involved in either *R* gene‐ or *PRR*‐mediated immune signalling suggests that *PpE4*‐triggered cell death is possibly the consequence of plant recognition. However, this recognition is not mediated by either BAK1‐ or SOBIR1‐associated cell surface receptors, or by EDS1‐ or NDR1‐associated R proteins, because cell death was only slightly affected in *BAK1*‐silenced plants and not significantly affected in *SOBIR1*‐, *EDS1*‐ or *NDR1*‐silenced plants (Fig. S8). According to a previous study, PpE4 shows moderate sequence similarity to *P. sojae* effector Avh238 (Yang *et al*., 2017). Although they are significantly divergent in the C‐terminal region, both *PpE4* and *Avh238* trigger non‐specific cell death in various plants, but the cell death mechanism may be distinct, as different genes are responsible for the mediation of cell death induced by each effector (Yang *et al*., 2017). Different components of the PTI or ETI pathways have been reported to be specifically involved in cell death induced by different RXLR effectors, which indicates that there are distinct recognition mechanisms and complicated signalling pathways responsible for each effector (Bos *et al*., 2006; Lee *et al*., 2018; Oh *et al*., 2009; Wang H *et al*., 2017; Xiang *et al*., 2017; Yang *et al*., 2017; Yu *et al*., 2012). However, it is still unclear how these effectors are recognized in plants.

It is worthwhile to elucidate the cell death induction mechanisms of early‐induced RXLR effectors. Studies of the biological functions and host targets of RXLR effectors are conducive to the illumination of the pathogenic mechanisms and the development of disease control strategies against pathogens, such as *P. parasitica*, which have a broad host range.

## Experimental Procedures

### Plant and *Phytophthora* cultivation

*Arabidopsis thaliana* seeds were sterilized and sown on 0.8% agar plates containing half‐strength Murashige and Skoog nutrient solution, followed by a 1‐week incubation in a growth chamber, as described previously (Wang Y *et al*., 2011). The seedlings were then transferred to a matrix containing soil and vermiculite, and grown in a 22–25 °C climate chamber with a photoperiod of 14 h light, 10 h dark and 70% relative humidity for 4 weeks. *Nicotiana benthamiana*, tobacco and tomato seeds, and potato tubers, were routinely cultured in a matrix in a climate chamber for about 5–6 weeks under the same conditions as used for the growth of *Arabidopsis*. The *P. parasitica* strain and transformants were cultured on 5% (v/v) carrot juice agar (CA) medium with 0.01% (w/v) CaCO_3_ and 0.002% (w/v) β‐sitosterol for 4 days at 23 °C. Then, 5% CA plugs with fresh mycelia were cultured in carrot broth for 4 days. To produce sporangia, carrot broth was replaced with Petri solution [Ca(NO3)_2_, 0.4 g/L; KH_2_PO_4_, 0.15 g/L; Mg(NO_3_)_2_, 0.15 g/L; CaCl_2_, 0.06 g/L], and the culture was cultivated for another 5 days. Zoospores were released by chilling and recovery as described previously (Wang Y *et al*., 2011).

### Total RNA extraction and RT‐qPCR analyses

Total RNA of different samples was extracted using TRIzol reagent (Invitrogen, Carlsbad, CA, USA) and reverse transcribed using a PrimeScript™ RT Reagent Kit with gDNA Eraser (TaKaRa, Dalian, China) according to the product manuals. RT‐qPCR was performed using 5 μL of a 1 : 10 dilution of first‐strand cDNA and SYBR Green mix (CWBio, Beijing, China) on a QuantStudio™ 3 Real‐Time PCR System (Thermo Scientific, Waltham, MA, USA). Gene‐specific primers were designed online (http://sg.idtdna.com/PrimerQuest/Home/Index), and the specificity was examined by performing dissociation curve assays. The previously described internal controls were chosen as follows: ubiquitin‐conjugating enzyme (*PpUBC*) and 40S ribosomal protein S3A (*PpWS21*) genes for *P. parasitica* (Yan and Liou, 2006); the *AtUBC9* gene for *A. thaliana*; and the β‐actin gene for *N. benthamiana*. For the biomass assay, primers specific to *PpUBC*, *AtUBC9* and *NbF*‐*box* were used for qPCR.

### Vector construction

All the primers and vectors used in this study are listed in Table S1 (see Supporting Information). The gene fragments were amplified using PrimeStar polymerase (TaKaRa) and digested using appropriate restriction endonucleases (Promega, Madison, WI, USA), followed by ligation into vectors using T4 DNA ligase (Promega). The *PpE4* and *GFP* co‐silencing hairpin vector pTH210::E4S was constructed with reference to a previous study (Meng *et al*., 2015). First, the *Spe*I‐ and *Cla*I‐digested *GFP* fragment and *Cla*I‐digested kanamycin resistance gene linker were ligated into *Spe*I‐linearized pBluescript II KS to generate the GFP‐linker‐GFP hairpin structure. Then, the *BamH*I‐ and *Spe*I‐digested *PpE4* fragment and the *Spe*I‐released GFP‐linker‐GFP fragment were ligated into *BamH*I‐linearized pBluescript II KS to generate the PpE4‐GFP‐linker‐GFP‐PpE4 co‐silencing hairpin structure. Finally, the co‐silencing hairpin structure, which was blunt‐ended by *Pfu* DNA Polymerase (Promega) after being digested by *BamH*I, was inserted into *Sma*I‐linearized plasmid pTH210 (Judelson *et al*., 1991). To construct the overexpression vector, *E4FL* was inserted into pMCherryH (*Ham34* promoter) after being digested with *Age*I and *Nhe*I, or fused with *mCherry* by overlapping PCR and then inserted into pTH210 (*Hsp70* promoter) after being digested with *Apa*I and *Kpn*I. The signal peptide of PpE4 was predicted using the SignalP 4.1 online server (http://www.cbs.dtu.dk/services/SignalP/) (Nielsen, 2017). *E4FL*, mature *PpE4* without a signal peptide and its deletion mutants were ligated into pCAMBIA1307‐3×Flag and pER8 vector. For the VIGS assay, primers were designed with reference to previous studies: *EDS1* and *RAR1* (Liu *et al*., 2002); *SGT1*, *HSP90* and *NPK* (Jin *et al*., 2002); *NDR1*, *MEK2*, *SIPK*, *MEK1*, *MYB1* and *WRKY3* (Liu *et al*., 2004b); *BAK1 *(Yang *et al*., 2017); and *SOBIR1* (Liebrand *et al*., 2013). Fragments amplified from *N. benthamiana* cDNA were cloned into the binary vector pTRV2. All constructs were sequenced by Genscript (Nanjing, China).

### *Agrobacterium tumefaciens*‐mediated transient expression

*Agrobacterium tumefaciens* GV3101 strains carrying the respective constructs were cultured in Luria–Bertani medium supplemented with the appropriate antibiotics at 28 °C for 1 day, and then harvested and suspended in infiltration buffer [10 mM 2‐(*N*‐morpholine)‐ethane sulfonic acid (MES), 10 mM MgCl_2_, pH 5.6, and 200 μM acetosyringone] to an appropriate concentration. For the inoculation of *P. parasitica* after transient expression, infiltrations were performed at a final OD_600_ of 0.01; otherwise, an OD_600_ value of 0.4 was used. After incubation for 1 h at 28 °C, the *A. tumefaciens* suspensions were infiltrated into plant leaves using needleless syringes (Meng *et al*., 2015). Cell death was observed at 3–5 dpi in *N. benthamiana* and tobacco species, and at 5–8 dpi in *Solanum lycopersicum* and *S. tuberosum*. For western blot analysis, proteins were extracted at 2 dpi. All experiments were repeated at least three times.

### VIGS assay in *N. benthamiana*

*Agrobacterium tumefaciens* GV3101 strains carrying different pTRV2 constructs were mixed with pTRV1 in equal ratios to a final OD_600_ of 0.25. pTRV2::*GFP* was used as a control, and pTRV2::*PDS* was used to visualize the silencing process. The lower leaves of four‐leaf stage *N. benthamiana* plants were infiltrated as described previously (Liu *et al*., 2002; Ratcliff *et al*., 2001), and the degree of cell death and gene silencing efficiency were analysed in the upper leaves at 16–20 dpi.

### Transformation of *A. thaliana*

*Agrobacterium tumefaciens* carrying the empty vector pER8 or pER8::3×*Flag*‐*PpE4* was cultured and suspended in a solution of 5% sucrose and 0.02% Silwet L‐77 (GE Healthcare, Uppsala, Sweden). *Arabidopsis* ecotype Col‐0 was transformed by dipping in the suspension as described previously (Clough and Bent, 1998). The kanamycin‐resistant seedlings were screened on selective medium and planted in soil. Then, the expression level of *PpE4* in transgenic plants after induction by 17‐β‐estradiol was determined by semi‐quantitative PCR (Zuo *et al*., 2000).

### Transformation of *P. parasitica*

To generate silencing and overexpressing transformants, *P. parasitica* protoplasts were transformed using the PEG–CaCl_2_‐mediated method as described previously (Bottin *et al*., 1999; Meng *et al*., 2015). The silencing and overexpression plasmids (pTH210::E4S, pMCherryH::*E4FL* and pTH210::*E4FL*‐*mCherry*) were linearized by *BamH*I and separately co‐transformed with linearized pTH209 into protoplasts of strain 1121, which stably expresses hyphal cytoplasmic GFP. The transformed protoplasts were regenerated overnight, and the recovered mycelia were selected on 5% CA medium with 4 µg/mL geneticin and 100 µg/mL hygromycin. After 3–7 days, the primary transformants were transferred to new selective medium in six‐well plates and named sequentially and maintained for subsequent analyses.

### Fluorescence microscopy

To identify *PpE4* and *GFP* co‐silencing transformants, transformants with attenuated GFP signal were identified using an Olympus BX‐51TRF fluorescence microscope (Olympus, Tokyo, Japan) with the GFP filter (BP450‐480). For *E4FL*‐*mCherry*‐expressing lines, the putative transformants were observed under the mCherry filter (BP520‐550). Images of vegetative mycelia and infection hyphae in *N. benthamiana* leaves were captured on an Olympus IX83‐FV1200 confocal microscope with 488 nm excitation and a 500–530 nm emission spectrum for GFP. For mCherry, the emission spectrum was acquired between 595 and 625 nm under 559 nm excitation to eliminate potential autofluorescence from *P. parasitica* hyphae and cell damage. The detached *N. benthamiana* leaves inoculated with the transformants and the control strain were incubated at 23 °C for 12–48 h to allow the penetration and formation of intercellular hyphae with haustoria. The control strain and transformants were observed under the same conditions.

### Inoculation of *P. parasitica*

*Nicotiana benthamiana* leaves were detached 24 h after agroinfiltration and kept in a plastic tray covered with moist filter paper. The petioles were wrapped with wet cotton; the leaves were inoculated with 1000 zoospores of *P. parasitica* strain 1121 and incubated in a growth chamber at 23 °C. For pathogenicity assays of *P. parasitica* transformants, fresh mycelia of transformants and the control strain grown on 5% CA plugs were inoculated on each side of detached *N. benthamiana* leaves. More than 15 leaves were used in each assay. At 36–48 hpi, the hyphal expansion was marked under a fluorescence microscope to measure the lesion diameter. Total DNA was extracted from identical areas on each side of the leaf, and the biomass was calculated by the DNA ratio of *P. parasitica* in infected tissues using qPCR (Meng *et al*., 2015). The rosette leaves of wild‐type *Arabidopsis* Col‐0 and T3 homozygous pER8 and pER8::3×*Flag*‐*PpE4* transgenic plants were injected with 10 μM 17‐β‐estradiol (Zuo *et al*., 2000). After 12 h, the treated leaves were detached and placed in a plastic tray with wet cotton covering the petioles. Then, 2000 zoospores were dropped onto the abaxial surface of each leaf. About 25 leaves from more than 15 plants of each line were analysed for each assay. The disease severity index (DSI) was recorded at 48 hpi, with grade 1 being no visible symptoms and few hyphae colonized on the leaf surface, grade 2 being the development of restricted water‐soaked lesions with a diameter of less than 2 mm, grade 3 being the development of water‐soaked lesions smaller than the inoculation sites with abundant hyphae colonized, and grade 4 being the development of large lesions with massive hyphae spreading beyond the inoculation sites. The expansion of *P. parasitica* hyphae was visualized by trypan blue staining and the *P. parasitica* biomass was determined in equal amounts of inoculated leaves by qPCR.

### Western blot analysis

Mycelia or plant leaves were ground into a powder in liquid nitrogen and vigorously mixed with a double volume of precooled RIPA lysis buffer [50 mM Tris (pH 7.4), 150 mM NaCl, 1% TritonX‐100, 1% sodium deoxycholate, 0.1% sodium dodecylsulfate (SDS), 5 mM sodium fluoride, 1 mM sodium orthovanadate, 1 mM ethylenediaminetetraacetic acid (EDTA), 10 mM dithiothreitol (DTT), 1% (w/v) protease inhibitor cocktail (Sigma, St. Louis, MO, USA)]. After 20 min of incubation on ice, the sample was centrifuged at 20 000 ***g*** for 15 min to obtain the supernatant. After the addition of loading buffer and boiling for 5 min, total proteins were separated by sodium dodecylsulfate‐polyacrylamide gel electrophoresis (SDS‐PAGE). Then, the proteins were transferred to poly(vinylidene difluoride) (PVDF) membranes (Roche, Basel, Switzerland), followed by blocking in 10% skimmed milk (BD, Sparks, MD, USA) dissolved in Tris‐buffered saline (TBS; pH 7.2). Mouse anti‐Flag monoclonal antibody (Abbkine, Redlands, CA, USA) and mouse anti‐mCherry monoclonal antibody (Abbkine) were used at 1 : 2000 dilution to detect the corresponding fusion proteins. The membranes were washed and incubated with a goat anti‐mouse antibody (Abbkine). The protein bands were visualized by chemiluminescence using an eECL Western blot kit (CWBio), and photographs were taken under a ChemiDOC™ XRS+ imaging system (Bio‐Rad Laboratories, Hercules, CA).

## Supporting information


**Fig. S1** ****Expression pattern of *PpE4* during *Phytophthora parasitica *infection of *Arabidopsis*. (A) FPKM (fragments per kilobase million) value of *PpE4* and other RXLR effector genes from RNA‐sequencing (RNA‐seq) data. The sums of the FPKM values of all the 76 RXLR effector genes detected (FPKM value larger than unity) during infection of *Arabidopsis* roots were calculated. (B) Relative *PpE4* transcript levels during different stages of *P. parasitica* infection quantified by reverse transcription‐quantitative polymerase chain reaction (RT‐qPCR). *Arabidopsis* roots inoculated with *P. parasitica* zoospores were harvested at different hours post‐inoculation (hpi). My, *P. parasitica* mycelia grown in carrot broth. The relative expression level of *PpE4* in mycelia was given a value of unity. Error bars represent the standard deviation (SD) of three pooled samples.

**Fig. S2** ****The infection process of *Phytophthora parasitica* on *Nicotiana benthamiana*. Biotrophic growth was dominant before 24 h post‐inoculation (hpi), followed by a rapid switch to necrotrophic growth with large‐scale cell death. *Nicotiana benthamiana* leaves infected with zoospores of strain 1121 [stably expresses hyphal cytoplasmic green fluorescent protein (GFP)] were observed under a fluorescence microscope at 3, 6, 12, 24 and 48 hpi. The green fluorescence represents infection hyphae; the red fluorescence is the chloroplast autofluorescence of healthy leaf cells, which turns black when cell death occurs in the leaves. At 3 hpi, the cysts germinated and colonized on the epidermal cells, and extensive hyphae formed at 6 hpi. Cell death occurred at the inoculation sites at 12 hpi. Together with the spread of abundant hyphae, cell death occurred at the whole inoculation sites at 24 hpi. At 48 hpi, cell death occurred in large areas, with sporangia developing at the inoculation sites. CD, cell death; S, sporangia. Bars, 100 μm.

**Fig. S3** ****Generation of *Phytophthora parasitica* transformants expressing the E4FL‐mCherry fusion protein. (A) Schematic diagram of the fusion protein constructs in vector pTH210 or pMCherryH. Expression of E4FL (full‐length PpE4 with its own signal peptide) fused with mCherry at its C‐terminus was driven by the constitutive *Ham34* or *Hsp70* promoter. Relative expression level of *PpE4* in vegetative mycelia (B) and in infected *Nicotiana benthamiana* leaves at 36 h post‐inoculation (hpi) (C) quantified by reverse transcription‐quantitative polymerase chain reaction (RT‐qPCR). Expression of E4FL‐mCherry in E4MC4A2 is driven by the *Ham34* promoter, whereas, in other transformants it is driven by the *Hsp70* promoter. The expression level of *PpE4* in strain 1121 was given a value of unity. Error bars represent the standard deviation (SD) of three biological replicates. (D) Accumulation of E4FL‐mCherry fusion proteins in vegetative mycelia was confirmed by western blot using mCherry antibody. Protein loading is indicated by Ponceau stain (PS). Similar results were obtained from three independent experiments.

**Fig. S4** ****The localization of E4FL‐mCherry in transformant E4MC4A6 during infection. (A) Confocal image showing the accumulation of E4FL‐mCherry outside the haustoria after secretion at 24 h post‐inoculation (hpi). (B) The fluorescence intensities of green fluorescent protein (GFP) and mCherry across the haustoria are indicated by the white lines labelled ‘1’ and ‘2’ in (A). Identical images were obtained from more than 10 haustoria in three independent biological replicates.

**Fig. S5** ****Attenuated pathogenicity of *E4FL‐mCherry*‐expressing *Phytophthora parasitica* transformants. Fresh mycelial plugs of transformants (E4MC4A2, E4MC4A6, E4MC4B2 and E4MC3N4) and control strain 1121 were inoculated on the left and right sides of *Nicotiana benthamiana* leaves, respectively, and the lesion diameters were measured at 48 h post‐inoculation (hpi). (A) Lesions caused by *E4FL‐mCherry*‐expressing transformants were significantly smaller than those caused by the 1121 strain and E4MC3N4. Error bars represent the standard deviation (SD) of 15 leaves. Asterisks denote significant differences from the control strain 1121 (two tailed *t*‐test: **P < 0.01; ***P < 0.001). (B) Representative inoculated leaves. Similar results were obtained from more than three independent experiments.

**Fig. S6** ****Generation of *Phytophthora parasitica PpE4*‐silencing transformants. (A) Diagram of the *PpE4* and *GFP* co‐silencing hairpin structure construct. The kanamycin‐resistant gene (*kanR*) was used as the linker sequence. (B) Green fluorescent protein (GFP) signals in mycelia of five *PpE4*‐silenced transformants and strain 1121. (C) Relative expression level of *PpE4* in five GFP signal‐decreased transformants sampled at 24 h post‐inoculation (hpi) on *Nicotiana benthamiana* leaves was quantified by reverse transcription‐quantitative polymerase chain reaction (RT‐qPCR). The expression level of *PpE4* in strain 1121 was given a value of unity. Error bars represent the standard deviation (SD) of three biological replicates. Two independent experiments were performed with similar results.

**Fig. S7** ****Secondary structure of the PpE4 protein predicted by Phyre2 (http://www.sbg.bio.ic.ac.uk/phyre2/html/page.cgi?xml:id=index).

**Fig. S8** *PpE4*‐triggered cell death is not compromised in *Nicotiana benthamiana* plants with silenced expression of several genes involved in plant immune signalling. *Nicotiana benthamiana* leaves were infiltrated with pTRV2 constructs targeting *EDS1*, *NDR1*, *MEK1*, *MEK2*, *SIPK*, *MYB1*, *WRKY3*, *EDS1*, *NDR1*, *BAK1* and *SOBIR1*; pTRV2::*GFP* was used as a control. *Agrobacterium tumefaciens* carrying *PpE4* was infiltrated into the upper leaves of silenced plants at 16–20 days post‐infiltration (dpi). (A) Cell death photographed at 5 dpi. (B) Quantification of cell death on *N. benthamiana* leaves. The degree of cell death was divided into three levels: no visible cell death, weak cell death and complete cell death. Asterisk represents a significant difference from the control (Wilcoxon rank‐sum test: *P < 0.05). (C) Relative expression levels of silenced genes in corresponding virus‐induced gene silencing (VIGS)‐treated plants determined by reverse transcription‐quantitative polymerase chain reaction (RT‐qPCR). Error bars represent the standard deviation (SD) of three biological replicates. The experiments were repeated three times with more than 10 plants for each TRV construct.

**Table S1** ****Primers and vectors used in this study.
