# Supplementary figures and images for: An RXLR effector secreted by Phytophthora parasitica is a virulence factor and triggers cell death in various plants
Source: Mol Plant Pathol. 2018 Nov 22;20(3):356–71. doi: 10.1111/mpp.12760 (PMC6637884; doi:10.1111/mpp.12760)

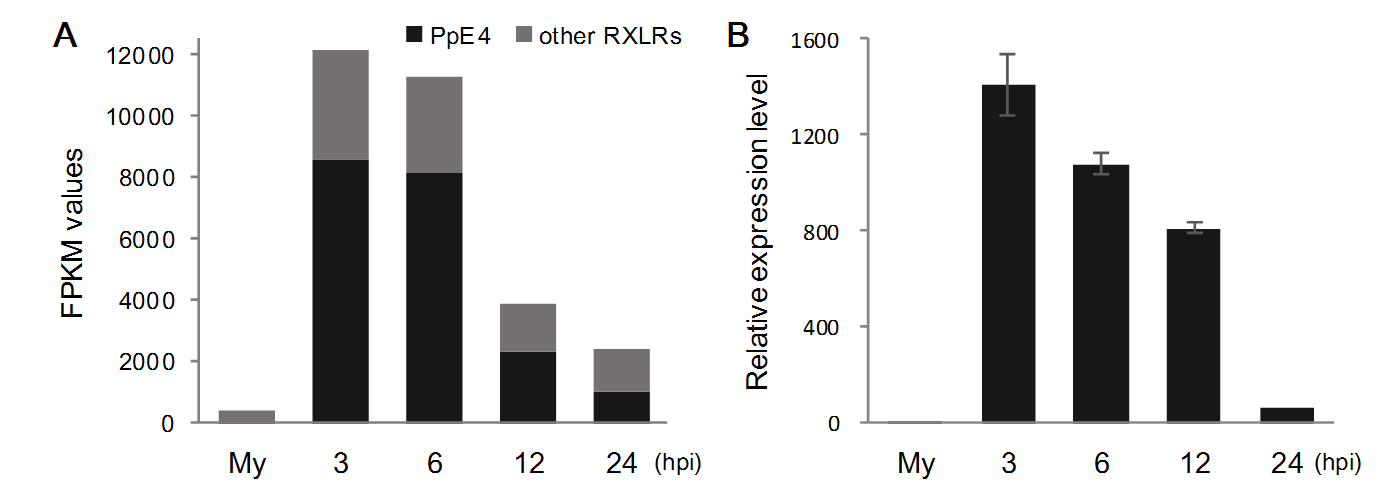

Supplement: Supplementary file 1 — Fig. S1 Expression pattern of PpE4 during Phytophthora parasitica infection of Arabidopsis. (A) FPKM (fragments per kilobase million) value of PpE4 and other RXLR effector genes from RNA‐sequencing (RNA‐seq) data. The sums of the FPKM values of all the 76 RXLR effector genes detected (FPKM value larger than unity) during infection of Arabidopsis roots were calculated. (B) Relative PpE4 transcript levels during different stages of P. parasitica infection quantified by reverse transcription‐quantitative polymerase chain reaction (RT‐qPCR). Arabidopsis roots inoculated with P. parasitica zoospores were harvested at different hours post‐inoculation (hpi). My, P. parasitica mycelia grown in carrot broth. The relative expression level of PpE4 in mycelia was given a value of unity. Error bars represent the standard deviation (SD) of three pooled samples. [file MPP-20-356-s001.tif]

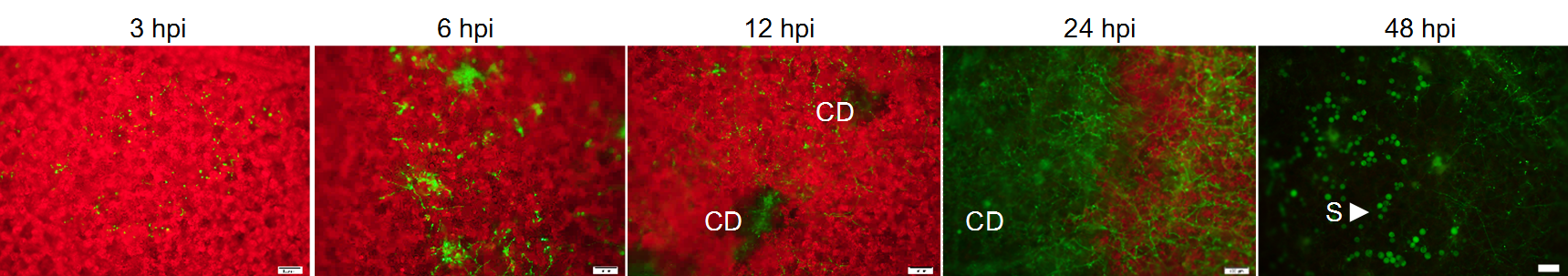

Supplement: Supplementary file 2 — Fig. S2 The infection process of Phytophthora parasitica on Nicotiana benthamiana. Biotrophic growth was dominant before 24 h post‐inoculation (hpi), followed by a rapid switch to necrotrophic growth with large‐scale cell death. Nicotiana benthamiana leaves infected with zoospores of strain 1121 [stably expresses hyphal cytoplasmic green fluorescent protein (GFP)] were observed under a fluorescence microscope at 3, 6, 12, 24 and 48 hpi. The green fluorescence represents infection hyphae; the red fluorescence is the chloroplast autofluorescence of healthy leaf cells, which turns black when cell death occurs in the leaves. At 3 hpi, the cysts germinated and colonized on the epidermal cells, and extensive hyphae formed at 6 hpi. Cell death occurred at the inoculation sites at 12 hpi. Together with the spread of abundant hyphae, cell death occurred at the whole inoculation sites at 24 hpi. At 48 hpi, cell death occurred in large areas, with sporangia developing at the inoculation sites. CD, cell death; S, sporangia. Bars, 100 μm. [file MPP-20-356-s002.tif]

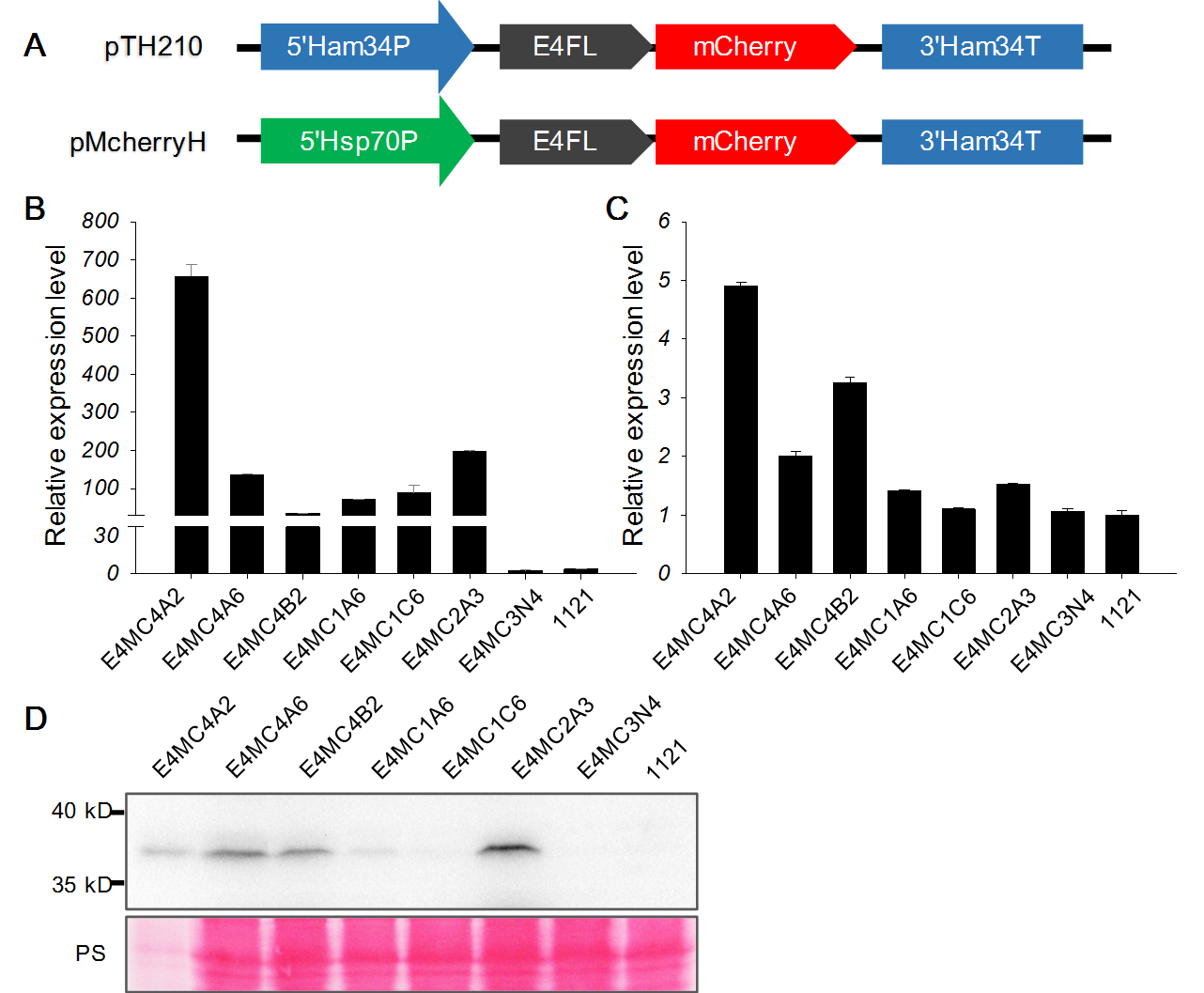

Supplement: Supplementary file 3 — Fig. S3 Generation of Phytophthora parasitica transformants expressing the E4FL‐mCherry fusion protein. (A) Schematic diagram of the fusion protein constructs in vector pTH210 or pMCherryH. Expression of E4FL (full‐length PpE4 with its own signal peptide) fused with mCherry at its C‐terminus was driven by the constitutive Ham34 or Hsp70 promoter. Relative expression level of PpE4 in vegetative mycelia (B) and in infected Nicotiana benthamiana leaves at 36 h post‐inoculation (hpi) (C) quantified by reverse transcription‐quantitative polymerase chain reaction (RT‐qPCR). Expression of E4FL‐mCherry in E4MC4A2 is driven by the Ham34 promoter, whereas, in other transformants it is driven by the Hsp70 promoter. The expression level of PpE4 in strain 1121 was given a value of unity. Error bars represent the standard deviation (SD) of three biological replicates. (D) Accumulation of E4FL‐mCherry fusion proteins in vegetative mycelia was confirmed by western blot using mCherry antibody. Protein loading is indicated by Ponceau stain (PS). Similar results were obtained from three independent experiments. [file MPP-20-356-s003.tif]

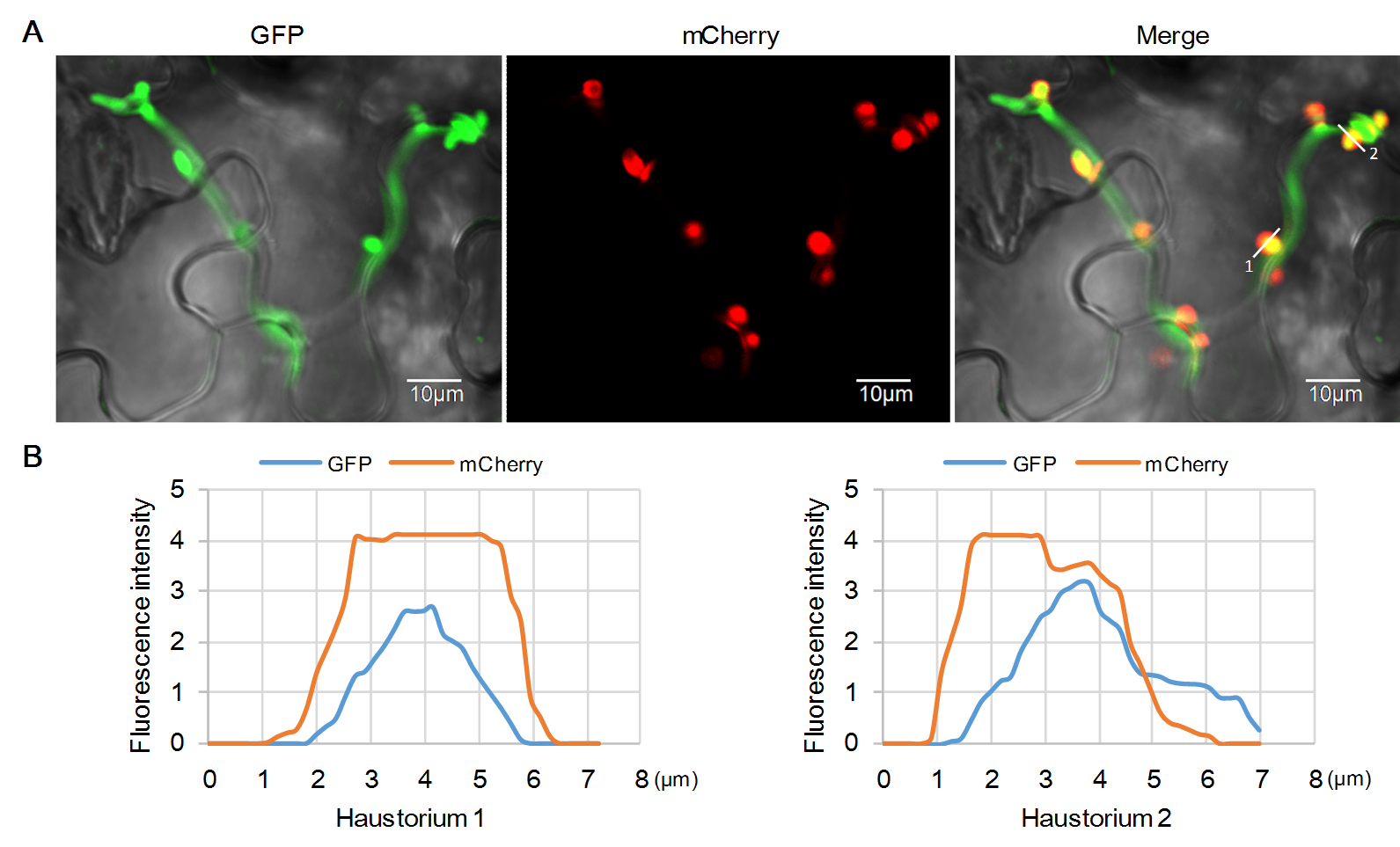

Supplement: Supplementary file 4 — Fig. S4 The localization of E4FL‐mCherry in transformant E4MC4A6 during infection. (A) Confocal image showing the accumulation of E4FL‐mCherry outside the haustoria after secretion at 24 h post‐inoculation (hpi). (B) The fluorescence intensities of green fluorescent protein (GFP) and mCherry across the haustoria are indicated by the white lines labelled ‘1’ and ‘2’ in (A). Identical images were obtained from more than 10 haustoria in three independent biological replicates. [file MPP-20-356-s004.tif]

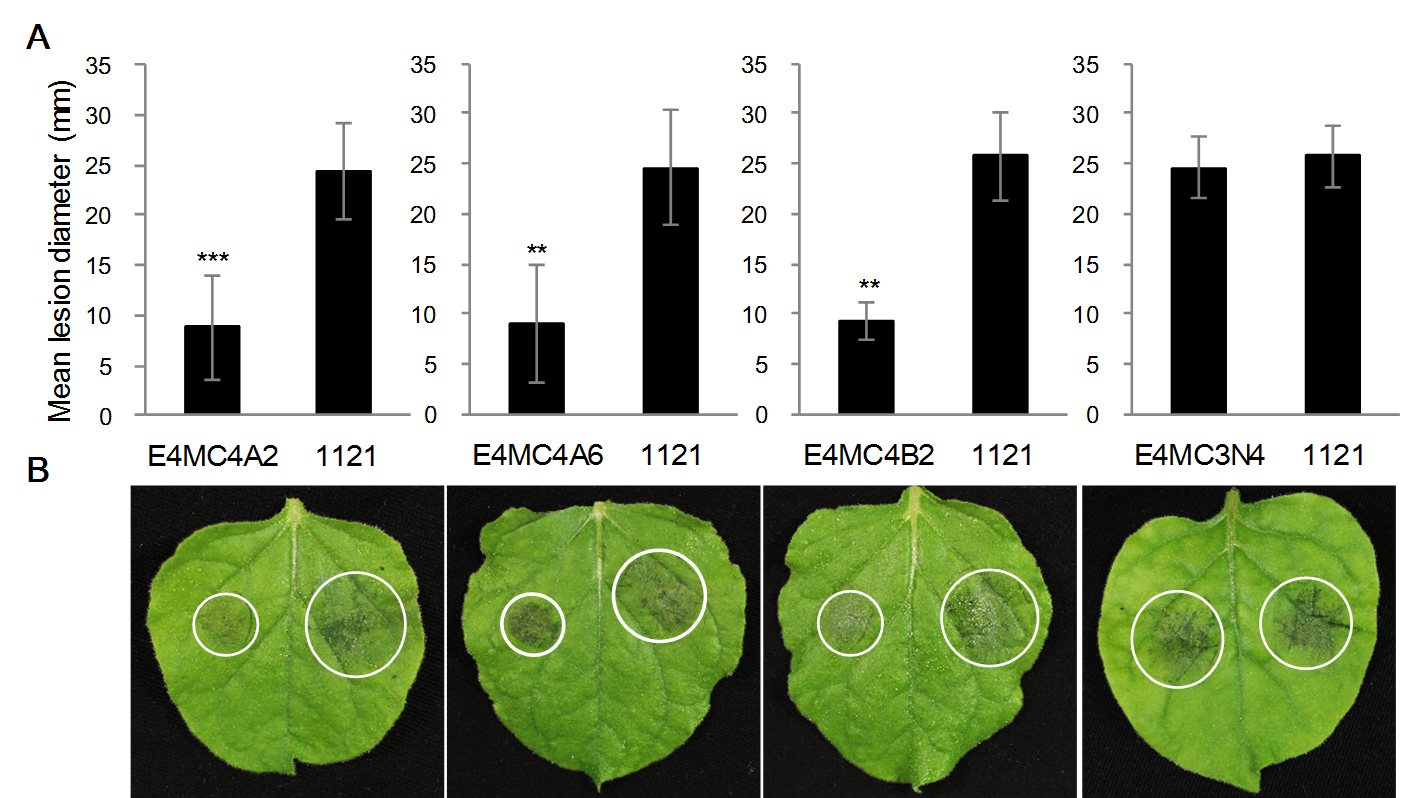

Supplement: Supplementary file 5 — Fig. S5 Attenuated pathogenicity of E4FL‐mCherry‐expressing Phytophthora parasitica transformants. Fresh mycelial plugs of transformants (E4MC4A2, E4MC4A6, E4MC4B2 and E4MC3N4) and control strain 1121 were inoculated on the left and right sides of Nicotiana benthamiana leaves, respectively, and the lesion diameters were measured at 48 h post‐inoculation (hpi). (A) Lesions caused by E4FL‐mCherry‐expressing transformants were significantly smaller than those caused by the 1121 strain and E4MC3N4. Error bars represent the standard deviation (SD) of 15 leaves. Asterisks denote significant differences from the control strain 1121 (two tailed t‐test: **P < 0.01; ***P < 0.001). (B) Representative inoculated leaves. Similar results were obtained from more than three independent experiments. [file MPP-20-356-s005.tif]

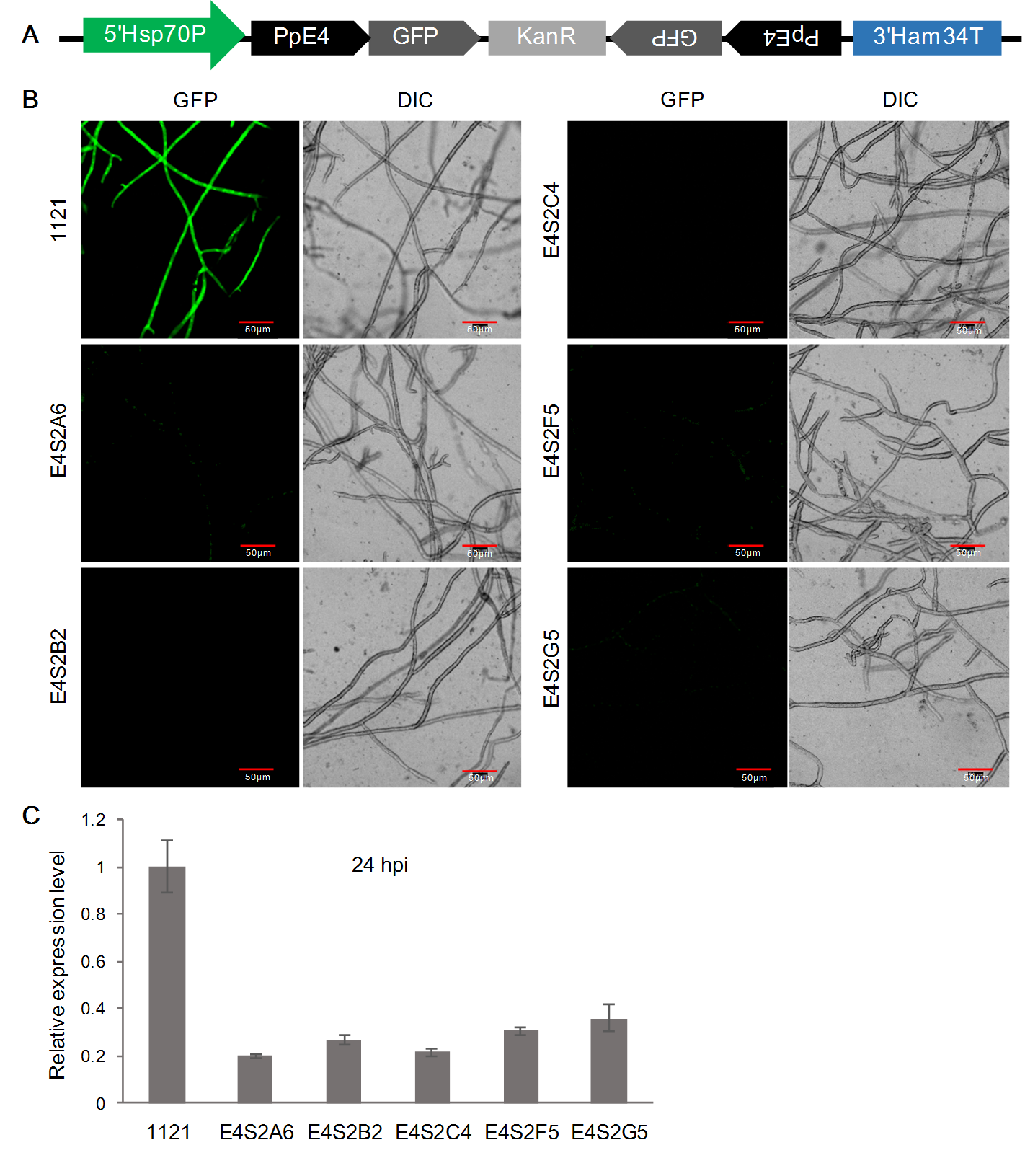

Supplement: Supplementary file 6 — Fig. S6 Generation of Phytophthora parasitica PpE4‐silencing transformants. (A) Diagram of the PpE4 and GFP co‐silencing hairpin structure construct. The kanamycin‐resistant gene (kanR) was used as the linker sequence. (B) Green fluorescent protein (GFP) signals in mycelia of five PpE4‐silenced transformants and strain 1121. (C) Relative expression level of PpE4 in five GFP signal‐decreased transformants sampled at 24 h post‐inoculation (hpi) on Nicotiana benthamiana leaves was quantified by reverse transcription‐quantitative polymerase chain reaction (RT‐qPCR). The expression level of PpE4 in strain 1121 was given a value of unity. Error bars represent the standard deviation (SD) of three biological replicates. Two independent experiments were performed with similar results. [file MPP-20-356-s006.tif]

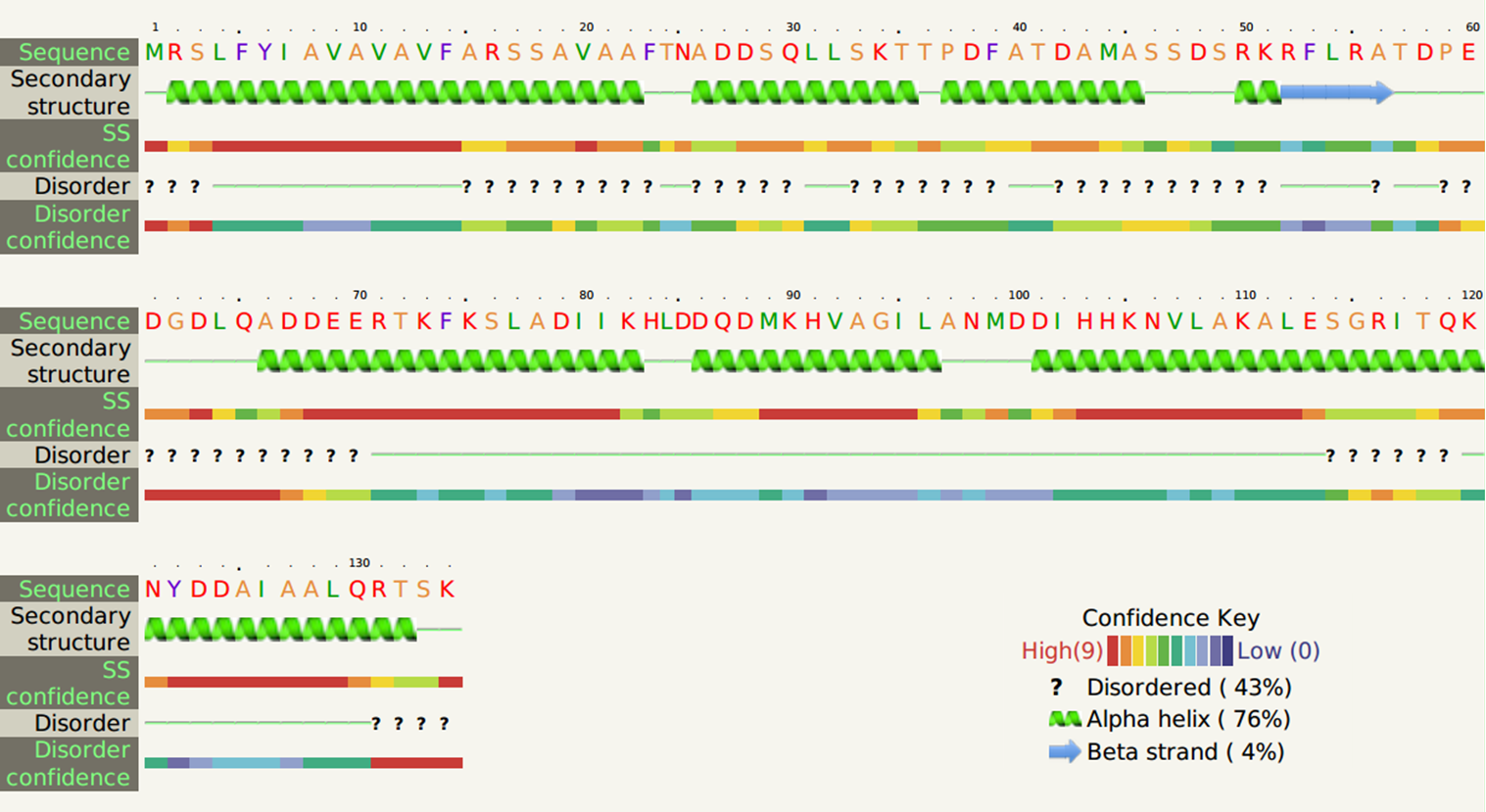

Supplement: Supplementary file 7 — Fig. S7 Secondary structure of the PpE4 protein predicted by Phyre2 (http://www.sbg.bio.ic.ac.uk/phyre2/html/page.cgi?xml:id=index). [file MPP-20-356-s007.tif]

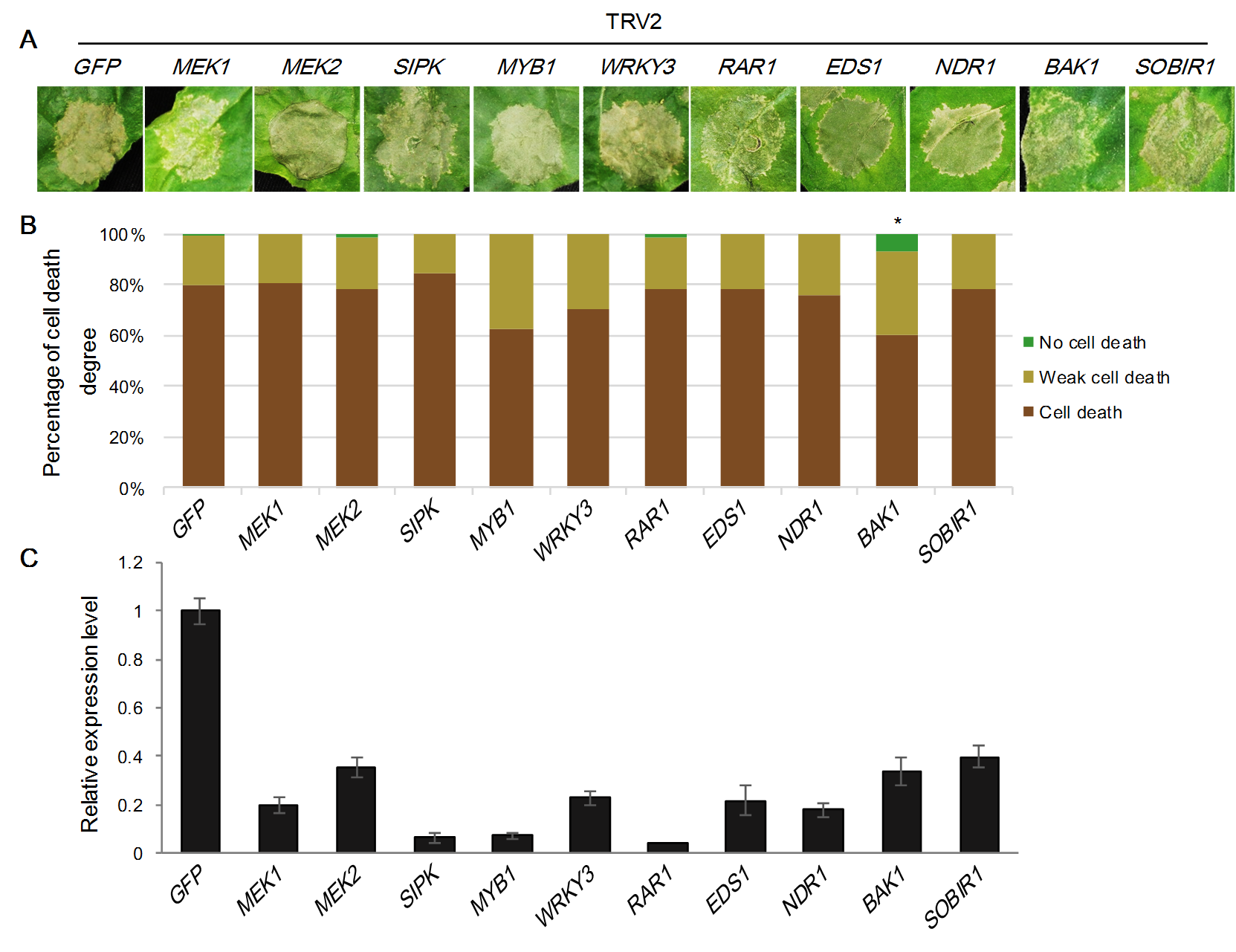

Supplement: Supplementary file 8 — Fig. S8 PpE4‐triggered cell death is not compromised in Nicotiana benthamiana plants with silenced expression of several genes involved in plant immune signalling. Nicotiana benthamiana leaves were infiltrated with pTRV2 constructs targeting EDS1, NDR1, MEK1, MEK2, SIPK, MYB1, WRKY3, EDS1, NDR1, BAK1 and SOBIR1; pTRV2::GFP was used as a control. Agrobacterium tumefaciens carrying PpE4 was infiltrated into the upper leaves of silenced plants at 16–20 days post‐infiltration (dpi). (A) Cell death photographed at 5 dpi. (B) Quantification of cell death on N. benthamiana leaves. The degree of cell death was divided into three levels: no visible cell death, weak cell death and complete cell death. Asterisk represents a significant difference from the control (Wilcoxon rank‐sum test: *P < 0.05). (C) Relative expression levels of silenced genes in corresponding virus‐induced gene silencing (VIGS)‐treated plants determined by reverse transcription‐quantitative polymerase chain reaction (RT‐qPCR). Error bars represent the standard deviation (SD) of three biological replicates. The experiments were repeated three times with more than 10 plants for each TRV construct. [file MPP-20-356-s008.tif]
