# Supplementary material for: An RXLR effector secreted by Phytophthora parasitica is a virulence factor and triggers cell death in various plants
Source: Mol Plant Pathol. 2018 Nov 22;20(3):356–71. doi: 10.1111/mpp.12760 (PMC6637884; doi:10.1111/mpp.12760)
Supplement: Supplementary file 9 — Table S1 Primers and vectors used in this study. [file MPP-20-356-s009.docx]

Table S1 Primers and vectors used in this study.

| Purpose | Plasmid | Primer Name | Primer Sequence 5'-3' | Restriction Enzyme |
| --- | --- | --- | --- | --- |
| PpE4 silencing | pTH210::E4S | PpE4S-F | CGCGGATCCCGATGATGAAGAGCGAACTAAG | *Bam*HI |
|  |  | PpE4S-R | GGACTAGTCTTTGACGTGCGTTGTAGAG | *Spe*I |
| E4FL-mCherry overexpression | pTH210::E4FL-mCherry | PpE4FL-F | CGCGGATCCCGGGCCCATGCGCAGTTTGTTCTACATTG | *Apa*I |
|  |  | PpE4_mC-R | CCTTGCTCACCATCTTTGACGTGCGTTGTAGAG |  |
|  |  | mCherry-F4 | GCACGTCAAAGATGGTGAGCAAGGGCGAGGA |  |
|  |  | mCherry-R | GGGGTACCCTACTTGTACAGCTCGTCCATG | *Kpn*I |
|  | pMCherryH:PpE4 | E4MC-F | AGCTACCGGTATGCGCAGTTTGTTCTACATTG | *Age*I |
|  |  | E4MC-R | CTAGCTAGCCTTTGACGTGCGTTGTAGAG | *Nhe*I |
| *Arabidopsis thaliana* transformation | pER8::3×Flag-PpE4 | 3Flag-2X-F | CCGCTCGAGTCTAGAATGGACTACAAAGACCATGATGGA | *Xho*I |
|  |  | PpE4-SpeI-R | GGACTAGTTTACTTTGACGTGCGTTGTAG | *Spe*I |
| Transient expression in plant | pCambia1307-3× Flag::PpE4 | attB1-PpE4-F | AGGCTTCGGATCCCATATGTTCACGAATGCCGACGATTC | *Bam*HI |
|  |  | attB2-PpE4-R2 | AAGCTGGGTCGACTCTAGATTACTTTGACGTGCGTTGTAGA | *Xba*I |
|  | pCambia1307-3× Flag::GFP | GFP6-F | CGCGGATCCATGAGTAAAGGAGAAGAACTTTTC | *Bam*HI |
|  |  | GFP6-R | GCTCTAGATTAGTGGTGGTGGTGGTGGT | *Xba*I |
|  | pCambia1307::E4FL-Flag | E4FL-NcoI | CATGCCATGGGCATGCGCAGTTTGTTCTACATTG | *Nco*I |
|  |  | PpE4Flag-R | GGACTAGTTCACTTATCATCATCATCCTTATAATCCTTTGACGTGCGTTGTAGAG | *Spe*I |
| PpE4 deletion mutants cloned into vector pCambia1307-3×Flag for cell death activity assay | E4M1 (46-134) | E4M1-F | AGGCTTCGGATCCCATATGAGTAGTGACTCCCGGAAGA | *Bam*HI |
|  |  | E4M1-R | AAGCTGGGTCGACTCTAGATTACTTTGACGTGCGTTGTAGA | *Xba*I |
|  | E4M3 (83-134) | E4M3-F | CGCGGATCCCACCTGGACGACCAAGATATG | *Bam*HI |
|  |  | E4M3-R | AAGCTGGGTCGACTCTAGATTACTTTGACGTGCGTTGTAGA | *Xba*I |
|  | E4M4 (23-121) | E4M4-F | AGGCTTCGGATCCCATATGTTCACGAATGCCGACGATTC | *Bam*HI |
|  |  | E4M4-R | GCTCTAGATTAGTTCTTCTGCGTGATTCGAC | *Xba*I |
|  | E4M5 (23-100) | E4M5-F | AGGCTTCGGATCCCATATGTTCACGAATGCCGACGATTC | *Bam*HI |
|  |  | E4M5-R | AAGCTGGGTCGACTCTAGATTAATCCATGTTCGCTAAAATGCC | *Xba*I |
|  | E4M8 (56-121) | E4M8-F | CGCGGATCCGCCACTGATCCGGAAGATG | *Bam*HI |
|  |  | E4M4-R | GCTCTAGATTAGTTCTTCTGCGTGATTCGAC | *Xba*I |
| VIGS assay in *Nicotiana benthamiana* | TRV2::NbBAK1 | NbBAK1-EcoRI-F | GGAATTCGCCCTTAACTGGGCAACG | *Eco*RI |
|  |  | NbBAK1-*BamH*I-R | CGGGATCCTGAGGGTGGTGAGCAGGA | *Bam*HI |
|  | TRV2::NbSOBIR1 | NbSOBIR1-EcoRI-F | GGAATTCAATCTTTATCCACCAGATCATGC | *Eco*RI |
|  |  | NbSOBIR1-*BamH*I-R | CGGGATCCCAGAAAGTTTTCCAATGGCAG | *BamH*I |
|  | TRV2::GFP | TRV2GFP-F | CGGAATTCTGCTGAAGTCAAGTTTGAGGGA | *Eco*RI |
|  |  | TRV2GFP-R | TCCCCCGGGATGATCAGCGAGTTGCACGC | *Sma*I |
|  | TRV2::NbHSP90 | NbHsp90F | CGGAATTCATCAACACTTTCTACAGCAAC | *Eco*RI |
|  |  | NbHsp90R | TCCCCCGGGACCAGCTTGAGATTCCCAGAC | *Sma*I |
|  | TRV2::NbSGT1 | NbSGT1F | CGGAATTCTCGCCGTTGACCTGTACACTCAAGC | *Eco*RI |
|  |  | NbSGT1R | TCCCCCGGGGCAGGTGTTATCTTGCCAAACAACCTAGG | *Sma*I |
|  | TRV2::NbRAR1 | NbRAR1F | CGGAATTCAGGAAAGCACACAACAGAAAAACC | *Eco*RI |
|  |  | NbRAR1R | TCCCCCGGGGTGCCATCCTTTGGTGCATGGAGG | *Sma*I |
|  | TRV2::NbEDS1 | NbEDS1F | CGGAATTCGAGTACCAGACCAAGTGTGATA | *Eco*RI |
|  |  | NbEDS1R | TCCCCCGGGGCTGAGGTGGGAGTGTTTTCCACC | *Sma*I |
|  | TRV2::NbNDR1 | NbNDR1F | CGGAATTCTGAACACCAAGATGAAGGACAA | *Eco*RI |
|  |  | NbNDR1R | TCCCCCGGGCAAAAGAAGCAAGGTGAATAAA | *Sma*I |
|  | TRV2::NbMEK1 | NbMEK1F | CGGAATTCTCAGTTCTCATTGGAAGATCTTGA | *Eco*RI |
|  |  | NbMEK1R | TCCCCCGGGGTAAAACCTGCTTGCAAACAACTGC | *Sma*I |
|  | TRV2::NbMEK2 | NbMEK2-1F | GATGGATCCATGCGACCTCTTCAACCACC | *Bam*HI |
|  |  | NbMEK2-1R | TCCCCCGGGCTCTCAAAGTCATCTACGGT | *Sma*I |
|  | TRV2::NbWRKY3 | NbWRKY3F | CGGAATTCATGAGAATGTCTGCAACAATGGA | *Eco*RI |
|  |  | NbWRKY3R | TCCCCCGGGTCAAGAAAACTCTATGGCCTCA | *Sma*I |
|  | TRV2::NbNPK | NbNPK1F | CGGAATTCGGTTGGAATACTTGCATAAGAATG | *Eco*RI |
|  |  | NbNPK1R | TCCCCCGGGCATCAGAGTTTCCTAGTTTCCAG | *Sma*I |
|  | TRV2::NbMYB | NbMYB1F | CGGAATTCGGTGAGAGCTCCTTGTTGTGAGA | *Eco*RI |
|  |  | NbMYB1R | TCCCCCGGGACTGGGCTATCAATGTGTTTTTG | *Sma*I |
|  | TRV2::NbSIPK | NbSIPKF1 | CGGAATTCTAAAGGTGCTTACGGCATCGTTTG | *Eco*RI |
|  |  | NbSIPKR1 | TCCCCCGGGTGGCGGATGTATCGTTTTGCATTC | *Sma*I |
| Quantitative PCR primers | PpE4 | PpE4q-F1 | TCATTAAGCACCTGGACGAC |  |
|  |  | PpE4q-R1 | TCGTCGTAGTTCTTCTGCGT |  |
|  |  | PpE4q-F4 | GATTTCTACGGGCCACTGAT |  |
|  |  | PpE4q-R4 | GTCGTCCAGGTGCTTAATGA |  |
|  | PpUBC | Ubc-F | GAGCCCTGCGTTGACTATCT |  |
|  |  | Ubc-R | GTACTTGGCGGTCCATTCG |  |
|  | PpWS21 | WS21-F | TACGCCAAGACGGCTCAGA |  |
|  |  | WS21-R | TTCCATCAGACGCACCAGG |  |
|  | NbF_box | F-Box_F | GGCACTCACAAACGTCTATTTC |  |
|  |  | F-Box_R | ACCTGGGAGGCATCCTGCTTAT |  |
|  | AtUBC9 | AtUBC9_F | CATCGGATAGCCCTTATTCTG |  |
|  |  | AtUBC9_R | TGGAACACCTTCGTCCTAAAA |  |
|  | NbActin | NbAct_F | ACCATCAATGATCGGAATGG |  |
|  |  | NbAct_R | GCTCATCCTATCAGCAATGC |  |
|  | NbBAK1 | NbBAK1q-F1 | CTGAACGGTTGCTTGTTTATCC |  |
|  |  | NbBAK1q-R1 | TACGCTTCCTTATTGACCACTC |  |
|  | NbSOBIR1 | NbSOBIR1q-F | CTTAGAAAAACTCTCTTTAGC |  |
|  |  | NbSOBIR1q-R | TATGGATTGGAGTGACATTATG |  |
|  | HSP90 | HSP90q-F1 | GTTTCAGCCTCGACGATCCCA |  |
|  |  | HSP90q-R1 | GTCAACATCAGCATCCCCAGAA |  |
|  |  | HSP90q-F2 | GTTATCGTGACCACAAAGCACAAT |  |
|  |  | HSP90q-R2 | CTACGTTCTTCAAGGTATTCCAGT |  |
|  |  | HSP90q-F3 | CTTCAGGTTTCAGTCTTGATGAG |  |
|  |  | HSP90q-R3 | GCAATCCTCATCGATACTCAGA |  |
|  | NPK | NPKq-F | ATGATGACATGTGCCAGATGGA |  |
|  |  | NPKq-R | TTATCAGGTTCACACATAGGGTT |  |
|  |  | NPKq-F1 | GAGCTTGAGGAAGAAGTGAATC |  |
|  |  | NPKq-R1 | GAGATTGAGCCACCAGGAACA |  |
|  | SGT1 | SGT1q-F1 | AGGACACCAGAAGAGCCATG |  |
|  |  | SGT1q-R1 | ACCTTCTTTGCACCGACTTCT |  |
|  | NDR1 | NDR1q-F | CCTGCCCTTAACAACTCTGATA |  |
|  |  | NDR1q-R | GAACACCTTTGTCCTTCATCTTG |  |
|  | EDS1 | EDS1q-F | GACTTAGGCCTGAGTACAAGAG |  |
|  |  | EDS1q-R1 | CTGTATCTTGCTTAATCCTTCCATG |  |
|  | MYB | MYBq-F | GATTACAAGCCTCCTCAGAACTC |  |
|  |  | MYBq-R | CAAAAACTCTCATCGATCTCTGG |  |
|  | SIPK | SIPKq-F | TACCTGAACTCGCTCCACGATAT |  |
|  |  | SIPKq-R | CCTTCATCTGTTCCTCCGTAAG |  |
|  | WRKY3 | WRKY3q-F | CGGAAGCCCAAAGATTCTACTT |  |
|  |  | WRKY3q-R | TTCTGCCCATATTTCCTCCATG |  |
|  | MEK1 | MEK1q-F | GGGCATGGTCATCCTTGAATG |  |
|  |  | MEK1q-R | ACAATAGCCTCCAGAAGCTCA |  |
|  | RAR1 | RAR1q-F | CATGATGGAATGAAGAAGTGGAG |  |
|  |  | RAR1q-R | AGCAGCTGACTTGGCTATCAC |  |
|  | MEK2 | MEK2q-F | GGACTTTATTGCTTGCTGTTTGC |  |
|  |  | MEK2q-R | CTATTCTGGGTAATAAATGGATGAC |  |
